# Uremic Toxin Receptor AhR Facilitates Renal Senescence and Fibrosis via Suppressing Mitochondrial Biogenesis

**DOI:** 10.1002/advs.202402066

**Published:** 2024-06-28

**Authors:** Hongyan Xie, Ninghao Yang, Li Lu, Xi'ang Sun, Jingyao Li, Xin Wang, Hengjiang Guo, Li Zhou, Jun Liu, Huijuan Wu, Chen Yu, Wei Zhang, Limin Lu

**Affiliations:** ^1^ Department of Physiology and Pathophysiology School of Basic Medical Sciences Fudan University Shanghai 200032 China; ^2^ Department of Nephrology Tongji Hospital Tongji University School of Medicine Shanghai 200065 China; ^3^ Department of Physiology and Pathophysiology School of Basic Medical Sciences Dali University Dali Yunnan 671013 China; ^4^ Department of Pathology School of Basic Medical Sciences Fudan University Shanghai 200032 China; ^5^ National Clinical Research Center for Aging and Medicine Huashan Hospital Fudan University Shanghai 200040 China; ^6^ Shanghai Kidney Development and Pediatric Kidney Disease Research Center Shanghai 201102 China

**Keywords:** aryl hydrocarbon receptor, cell senescence, mitochondrial biogenesis, PGC1α, renal fibrosis

## Abstract

Retention of metabolic end‐products in the bodily fluids of patients with chronic kidney disease (CKD) may lead to uremia. The uremic toxin indoxyl sulfate (IS), a tryptophan metabolite, is an endogenous ligand of aryl hydrocarbon receptor (AhR). It is clarified that the upregulation and activation of AhR by IS in tubular epithelial cells (TECs) promote renal senescence and fibrosis. Renal TEC‐specific knockout of *AhR* attenuates renal senescence and fibrosis, as well as the suppression of PGC1α‐mediated mitochondrial biogenesis in ischemia reperfusion (IR)‐ or IS‐treated CKD mice kidneys. Overexpression of peroxisome proliferator‐activated receptor gamma coactivator 1‐α (PGC1α) attenuates IS‐induced cell senescence and extracellular matrix production in cultured TECs. Mechanistically, AhR is able to interact with PGC1α and promotes the ubiquitin degradation of PGC1α via its E3 ubiquitin ligase activity. In summary, the elevation and activation of AhR by the accumulated uremic toxins in the progression of CKD accelerate renal senescence and fibrosis by suppressing mitochondrial biogenesis via promoting ubiquitination and proteasomal degradation of PGC1α.

## Graphical abstract

Uremic toxin receptor aryl hydrocarbon receptor (AhR) is upregulated following the accumulation of uremic toxins in the bodily fluids. The elevated AhR suppresses mitochondrial biogenesis by decreasing peroxisome proliferator‐activated receptor gamma coactivator 1‐α (PGC1α) via the E3 ubiquitin ligase activity. Insufficiency of mitochondrial function accelerates renal tubular epithelial cell senescence and fibrosis.

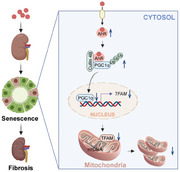

## 1 Introduction

Chronic kidney disease (CKD) affects nearly 10% of adults worldwide and is characterized by progressive decline in renal function and irreversible morphological changes. Understanding the mechanism of CKD is essential in nephrological research.^[^ ^1^ ^]^

Recent reports indicate the appearance of a senescent phenotype in the kidneys with interstitial fibrosis.^[^ ^2^, ^3^ ^]^ Senescent cells, such as the senescent tubular epithelial cells (TECs), that exhibit secretory phenotypes and produce proinflammatory and profibrotic cytokines, facilitate the progression of renal fibrosis.^[^ ^4^ ^]^ An opinion was recently accepted that renal fibrosis is a consequence of the accelerated senescence of the kidneys under pathological conditions, and senescence is an initiator of renal functional deterioration.^[^ ^2^, ^3^, ^4^, ^5^ ^]^ However, the exact mechanism that mediates renal senescence is unclear.

The kidney is a high‐energy‐consuming organ with abundant mitochondria.^[^ ^6^ ^]^ The mitochondrion is the dominant organelle that controls cellular energy metabolism.^[^ ^7^ ^]^ Disturbance in mitochondrial homeostasis has been broadly observed in nearly all renal disorders, especially aging‐relevant diseases.^[^ ^8^, ^9^, ^10^, ^11^ ^]^ Senescent cells have a decreased mitochondrial metabolic rate. Therefore, elucidating the mechanism of the disturbance in mitochondrial homeostasis could be meaningful for understanding the principles of CKD and exploring novel therapeutic strategies.

A decline in renal function impairs the renal excretion of metabolic end‐products (e.g., uremic toxins), which are thus retained in the bodily fluids.^[^ ^12^ ^]^ The retention of uremic toxins proves harmful to almost all organs and tissues. Recently, the accumulation of uremic toxins was noticed to accelerate renal senescence and deterioration,^[^ ^13^, ^14^ ^]^ but the underlying molecular mechanisms remain unclear.

Aryl hydrocarbon receptor (AhR) is primarily known as the sensor for detecting environmental toxins, such as 2,3,7,9‐tetrachlorodibenzodioxin.^[^ ^15^, ^16^ ^]^ Emerging evidence has shown that the uremic toxins indoxyl sulfate (IS) and kynurenine, which are metabolites of tryptophan, are endogenous ligands of AhR, so AhR is also considered a uremic toxin receptor.^[^ ^17^, ^18^ ^]^ AhR is canonically a ligand‐activated transcription factor. Followed by heterodimerization with AhR nuclear translocator, AhR undergoes ligand‐bound nuclear translocation and then initiates the transcription of target genes. Cytochrome P450 1A1 is a well‐known target gene.^[^ ^19^, ^20^ ^]^ A recently identified non‐canonical function of AhR is that AhR functions as a ligand‐dependent E3 ubiquitin ligase. AhR, together with damaged‐DNA binding protein 1, RING‐box protein 1, transducin‐β‐like protein 3 and scaffold protein cullin 4B, forms an E3 ubiquitin ligase complex. AhR acts as a substrate‐specific adaptor for the E3 ubiquitin ligase complex, while cullin 4B is the core enzymatic component. It has been reported that AhR regulates the proteasomal degradation of sex steroid receptors.^[^ ^21^ ^]^ AhR expression has been observed to be increased in the renal tubules of CKD and acute kidney injury mice, but the role of AhR elevation has not been fully investigated.^[^ ^19^, ^22^, ^23^ ^]^

In the present study, we presented data that AhR was elevated following the accumulation of uremic toxins. AhR promoted the degradation of peroxisome proliferator‐activated receptor gamma coactivator 1‐α (PGC1α) via its ubiquitin ligase activity. As a key regulator of mitochondrial biogenesis, the decrease in PGC1α suppressed mitochondrial biogenesis and adenosine triphosphate (ATP) production and thus facilitated renal senescence and fibrosis.

## 2 Results

### 2.1 AhR is Upregulated in Ischemia Reperfusion (IR)‐ or IS‐Treated Mice and Mouse Tubular Epithelial Cells (mTECs)

**Figure 1:**
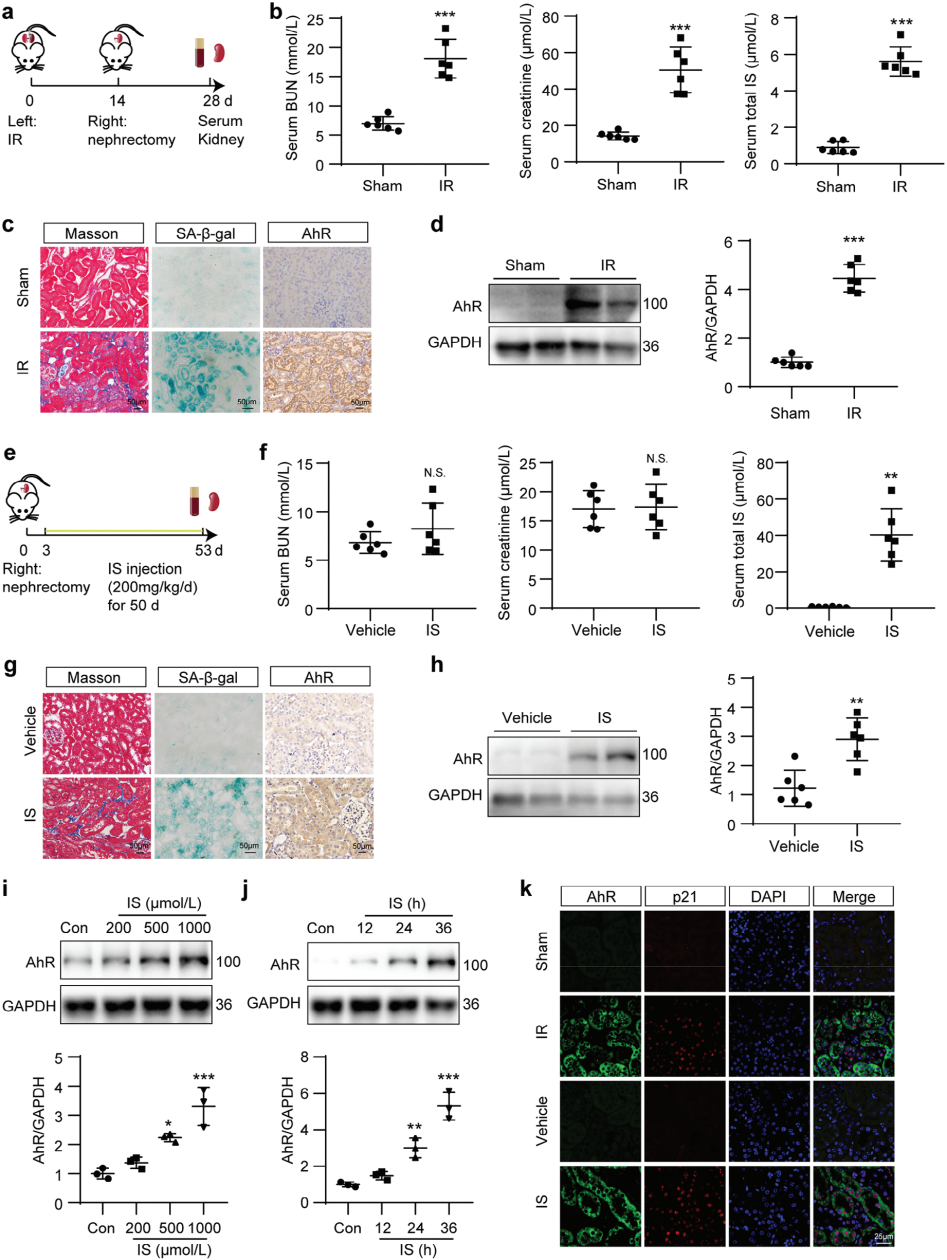
AhR was increased in the kidneys of IR‐ or IS‐treated mice. a) Schematic of IR mice preparation. b) Concentration of serum BUN, creatinine and IS in mice after IR surgery. ^***^ *P* < 0.001 versus sham group (*n *= 6). c) Masson, SA‐β‐gal staining and immunohistochemistry of AhR micrographs in mouse kidneys after IR surgery. Scale bar, 50 µm. d) Western blot images and quantitative analysis of AhR in mouse kidneys after IR surgery. ^***^ *P* < 0.001 versus sham group (*n *= 6). e) Schematic of IS‐treated mice preparation. f) Concentration of serum BUN, creatinine and IS in mice after IS treatment. ^**^ *P* < 0.01 versus vehicle group. N.S., no significant difference versus vehicle group (*n *= 6). g) Masson, SA‐β‐gal staining and immunohistochemistry of AhR micrographs in mouse kidneys after IS treatment. Scale bar, 50 µm. h) Western blot images and quantitative analysis of AhR in mouse kidneys after IS treatment. ^**^ *P* < 0.01 versus vehicle group (*n *= 6). i,j) Western bolt images and quantitative analysis of AhR in mouse tubular epithelial cells (mTECs) treated with the indicated concentrations of IS for prespecified durations. ^*^ *P* < 0.05, ^**^ *P* < 0.01, ^***^ *P* < 0.001 versus control (Con) group (*n *= 3). k) Co‐immunofluorescence of AhR and p21 in mouse kidneys after IR or IS treatment. Scale bar, 25 µm. Data were shown as mean ± SD. Statistical analysis was performed by two‐tailed unpaired Student's *t*‐test (the right panel of b, the left and middle panel of f, h), two‐tailed unpaired Welch’ *t*‐test (the left and middle panel of b, d, the right panel of f), and one‐way ANOVA with Tukey's multiple comparisons test (i, j).

In IR‐induced CKD mice, serum concentrations of blood urea nitrogen (BUN), creatinine and IS were apparently increased at 28 days after IR surgery (**Figure** **1**a,b). Masson and SA‐β‐gal staining results showed significant increases in the extracellular matrix (ECM) deposition and senescence‐associated β‐galactosidase (SA‐β‐gal)‐positive staining area. Immunohistochemical staining and Western blot results showed that AhR was increased and mainly located in the TECs within the kidneys of IR mice (Figure 1c,d).

To confirm the effect of uremic toxins and obviate the potential interference of other factors in the IR model, an IS‐induced CKD model was established (Figure 1e) as previously described.^[^ ^13^ ^]^ Biochemical analysis showed that serum IS was significantly elevated, whereas no obvious changes in serum BUN or creatinine were observed after 50‐day IS treatment (Figure 1f). Besides, ECM deposition, SA‐β‐gal‐positive staining area, and the expression of AhR were elevated in the kidneys of IS‐treated mice (Figure 1g,h). In cultured mTECs, the expression of AhR was increased in a concentration‐ and dose‐dependent manner after IS treatment (Figure 1i,j). Colocalization of AhR with p21, a marker of senescence, was increased in tubules after IR or IS treatment, which suggested that AhR was expressed in TECs with a senescent phenotype (Figure 1k). Besides, AhR was also colocalized with p16^INK4A^, another marker of senescence, and the colocalization signals were increased in renal tubules of IR and IS mice (Figure S1a, Supporting Information). Collectively, these results indicated that the uremic toxin receptor AhR was increased and colocalized with senescent TECs in fibrotic kidneys.

The mRNA level of *AhR* in IR and IS mouse kidneys was increased (Figure S1b,c, Supporting Information). In addition, we searched the Nephroseq database and analyzed *AhR* mRNA level in the kidneys of healthy individuals and CKD patients. The data showed that the *AhR* mRNA level was increased in the kidneys of patients with diabetic nephropathy (Figure S1d,e, Supporting Information) and CKD (Figure S1f, Supporting Information). The *AhR* mRNA level in the kidneys of patients with CKD was negatively correlated with the estimated glomerular filtration rate (eGFR; Figure S1g, Supporting Information).

### 2.2 TEC‐Specific Knockout of AhR Attenuated Renal Injury, Senescence, and Fibrosis in IR Mice

**Figure 2:**
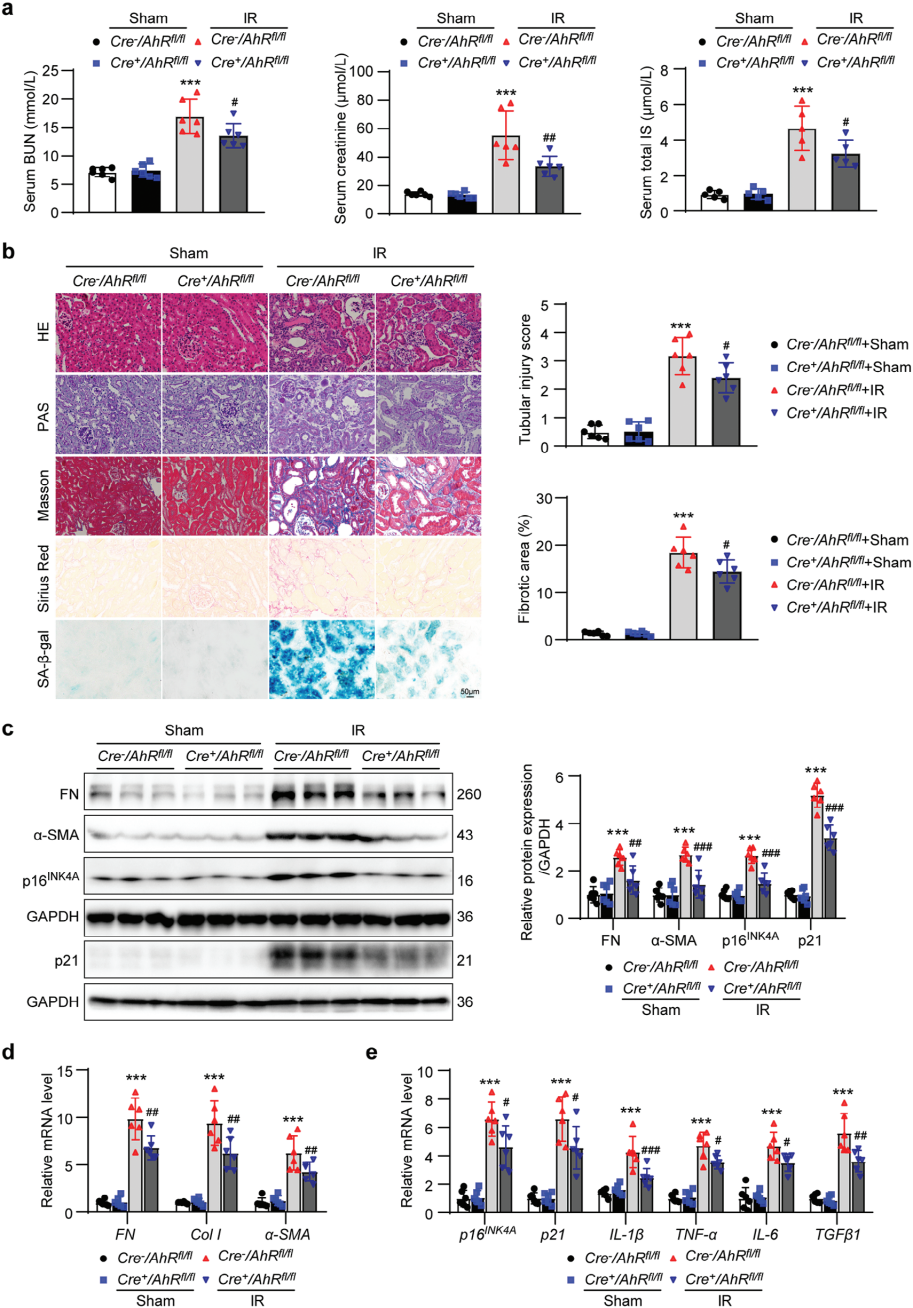
Renal tubular epithelial cell‐specific knockout of *AhR* mitigated the decline in renal function, senescence and fibrosis in IR mice. a) Serum BUN, creatinine and IS concentrations in mice from different groups. ^***^ *P* < 0.001 versus *Cre^−^AhR^fl/fl^* +sham group, ^#^ *P* < 0.05, ^##^ *P* < 0.01 versus *Cre^−^AhR^fl/fl^* +IR group (*n *= 5 or 6). b) HE, PAS, Masson, Sirius red and SA‐β‐gal staining micrographs of kidneys in mice from different groups. Scale bar, 50 µm. Evaluation of tubular injury score based on HE staining. Fibrotic area evaluation based on Masson staining. ^***^ *P* < 0.001 versus *Cre^−^AhR^fl/fl^* +sham group, ^#^ *P* < 0.05 versus *Cre^−^AhR^fl/fl^* +IR group (*n *= 6). c) Western blot images and quantitative analysis of fibrosis markers FN and α‐SMA, and senescence markers p16^INK4A^ and p21 in kidneys from different groups of mice. ^***^ *P* < 0.001 versus *Cre^−^AhR^fl/fl^* +sham group, ^##^ *P* < 0.01, ^###^ *P* < 0.001 versus *Cre^−^AhR^fl/fl^* +IR group (*n *= 6). d) The relative mRNA abundance of fibrosis markers *FN*, *Col I*, and *α‐SMA* in kidneys from different groups of mice. ^***^ *P* < 0.001 versus *Cre^−^AhR^fl/fl^* +sham group, ^##^ *P* < 0.01 versus *Cre^−^AhR^fl/fl^* +IR group (*n *= 6). e) The relative mRNA abundance of senescence markers *p16^INK4A^*, *p21*, and secretory factors *IL‐1β*, *TNF‐α*, *IL‐6*, and *TGFβ1* in kidneys from different groups of mice. ^***^ *P* < 0.001 versus *Cre^−^AhR^fl/fl^* +sham group, ^#^ *P* < 0.05, ^##^ *P* < 0.01, ^###^ *P* < 0.001 versus *Cre^−^AhR^fl/fl^* +IR group (*n *= 6). Data were shown as mean ± SD. Statistical analysis was performed by two‐way ANOVA with Tukey's multiple comparisons test (a‐e).

To investigate the role of AhR elevation in renal fibrosis, TEC‐specific *AhR* knockout mice (*Cre^+^AhR^fl/fl^*) were generated by crossbreeding transgenic *AhR^fl/fl^* mice with *Ggt1‐Cre* mice (Figure S2a, Supporting Information). The genotypes of the mice were identified by PCR (Figure S2b, Supporting Information), and *Cre^−^AhR^fl/fl^* littermates were used as controls. Western blot results showed that the expression of AhR was significantly reduced in the kidneys of *Cre^+^AhR^fl/fl^* mice when compared with *Cre^−^AhR^fl/fl^* mice (Figure S2c, Supporting Information). Immunofluorescence results showed that the colocalization signals of AhR and Megalin (a marker of proximal tubules) or NCC (a marker of distal tubules) were increased in IR‐ and IS‐treated *Cre^−^AhR^fl/fl^* mice. The signals of AhR disappeared in the proximal tubules, but not in the distal tubules of IR‐ and IS‐treated *Cre^+^AhR^fl/fl^* mice (Figure S2d,e, Supporting Information). Compared with *Cre^−^AhR^fl/fl^* mice, *Cre^+^AhR^fl/fl^* mice did not show obvious changes in renal function, histomorphology, or ECM production. However, the elevation in serum BUN, creatinine and IS induced by IR treatment was significantly blunted in *Cre^+^AhR^fl/fl^* mice when compared with *Cre^−^AhR^fl/fl^* mice (**Figure** **2**a). Hematoxylin and eosin (HE), periodic acid–Schiff (PAS), Masson and Sirius red staining results showed that the renal tubular injury and collagen deposition induced by IR treatment were also attenuated in *Cre^+^AhR^fl/fl^* mice. SA‐β‐gal staining showed that the increased SA‐β‐gal‐positive signals induced by IR treatment were alleviated in *Cre^+^AhR^fl/fl^* mice when compared with *Cre^−^AhR^fl/fl^* mice (Figure 2b). Western blot results showed that fibrosis markers fibronectin (FN) and α‐smooth muscle actin (α‐SMA), as well as senescence markers p16^INK4A^ and p21 were significantly elevated in *Cre^−^AhR^fl/fl^* mice after IR treatment, and these changes were blunted in *Cre^+^AhR^fl/fl^* mice (Figure 2c). Similar changes in *FN*, collagen I (*Col I*) and *α‐SMA* were confirmed by evaluating the mRNA levels (Figure 2d). Meanwhile, TEC‐specific knockout of *AhR* evidently inhibited the IR‐induced increases in the mRNA levels of *p16^INK4A^* and *p21*, and the secretory factors *IL‐1β*, *TNF‐α*, *IL‐6*, and *TGFβ1* (Figure 2e). These results suggested that TEC‐specific knockout of *AhR* attenuated IR‐induced renal dysfunction, tubular injury, interstitial ECM deposition, and the appearance of a senescent phenotype.

### 2.3 TEC‐Specific Knockout of AhR Attenuated Renal Injury, Senescence, and Fibrosis in IS‐Treated Mice

**Figure 3:**
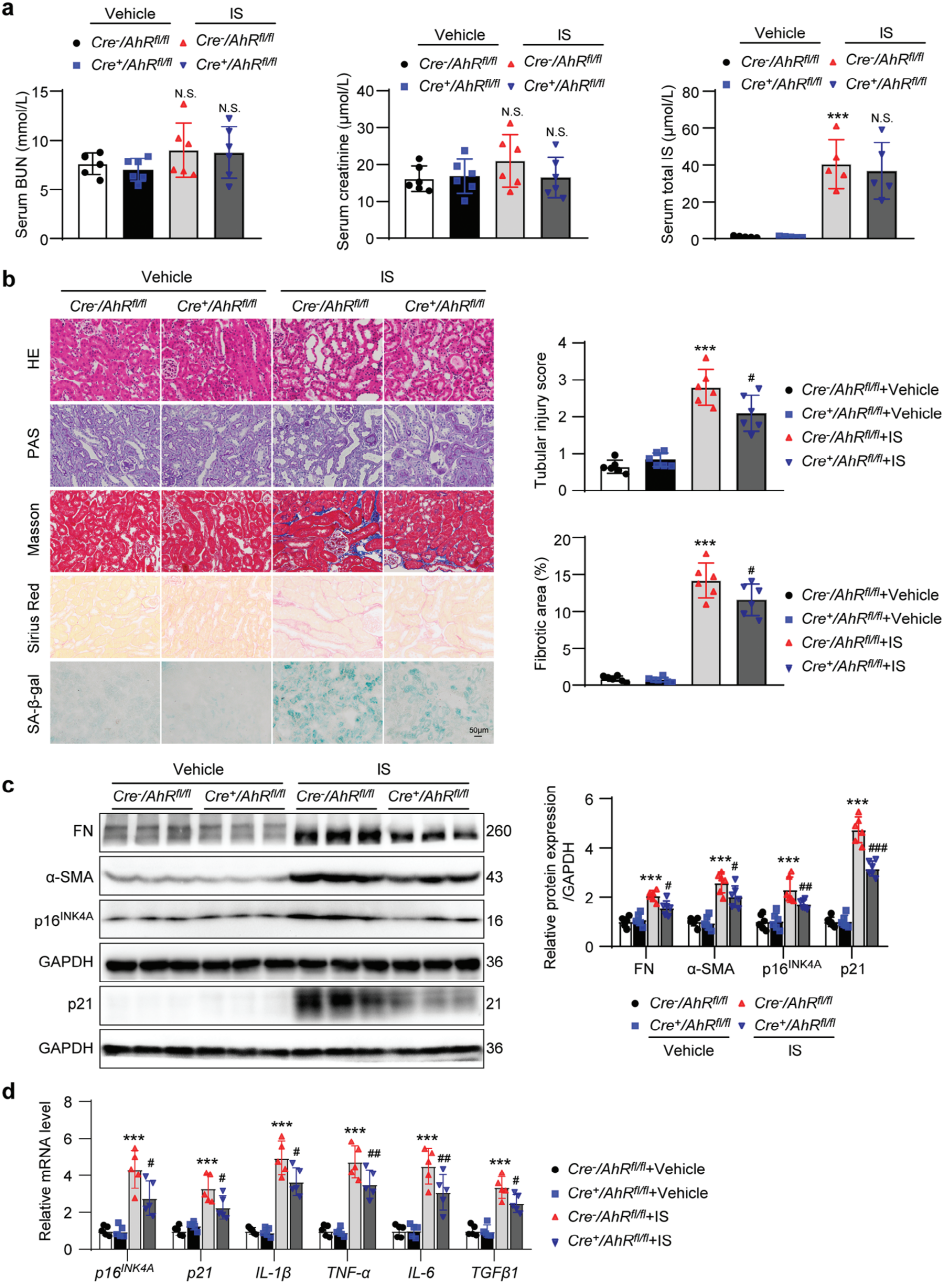
Renal tubular epithelial cell‐specific knockout of *AhR* mitigated the decline in renal function, senescence and fibrosis in IS‐treated mice. a) Serum BUN, creatinine and IS concentration in different groups of mice. ^***^ *P* < 0.001 versus *Cre^−^AhR^fl/fl^* +vehicle group. N.S., no significant difference versus *Cre^−^AhR^fl/fl^* +vehicle group or *Cre^−^AhR^fl/fl^* +IS group (*n *= 5 or 6). b) HE, PAS, Masson, Sirius red and SA‐β‐gal staining micrographs of kidneys from different groups of mice. Scale bar, 50 µm. Evaluation of tubular injury score based on HE staining. Fibrotic area evaluation based on Masson staining. ^***^ *P* < 0.001 versus *Cre^−^AhR^fl/fl^* +vehicle group, ^#^ *P* < 0.05 versus *Cre^−^AhR^fl/fl^* +IS group (*n *= 6). c) Western blot images and quantitative data showing FN, α‐SMA, p16^INK4A^, and p21 in kidneys from different groups of mice. ^***^ *P* < 0.001 versus *Cre^−^AhR^fl/fl^* +vehicle group, ^#^ *P* < 0.05, ^##^ *P* < 0.01, ^###^ *P* < 0.001 versus *Cre^−^AhR^fl/fl^* +IS group (*n *= 6). d) qPCR showing the mRNA levels of *p16^INK4A^*, *p21*, *IL‐1β*, *TNF‐α*, *IL‐6*, and *TGFβ1* in kidneys from different groups of mice. ^***^ *P* < 0.001 versus *Cre^−^AhR^fl/fl^* +vehicle group, ^#^ *P* < 0.05, ^##^ *P* < 0.01 versus *Cre^−^AhR^fl/fl^* +IS group (*n *= 5). Data were shown as mean ± SD. Statistical analysis was performed by two‐way ANOVA with Tukey's multiple comparisons test (a‐d).

To confirm the effects of AhR upregulation on tubular injury, renal senescence and fibrosis, *Cre^+^AhR^fl/fl^* mice were recruited in an IS‐induced CKD model. As shown in **Figure** **3**a, chronic IS administration significantly increased the serum IS level, albeit without obvious change in the serum BUN and creatinine levels. Compared with IS‐treated *Cre^−^AhR^fl/fl^* mice, no obvious difference in serum IS was observed in IS‐treated *Cre^+^AhR^fl/fl^* mice, which suggested that the knockout of *AhR* in renal TECs did not alter the elevated IS status in IS‐treated mice. HE, PAS, Masson and Sirius red staining showed that IS administration induced renal tubular injury and interstitial ECM sedimentation compared with vehicle‐treated mice. These changes were blunted in the IS‐treated *Cre^+^AhR^fl/fl^* mice. Besides, SA‐β‐gal staining showed that the increase in SA‐β‐gal‐positive signals induced by IS treatment was alleviated in *Cre^+^AhR^fl/fl^* mice when compared with *Cre^−^AhR^fl/fl^* mice (Figure 3b). The elevation in FN, α‐SMA, p16^INK4A^, and p21 analyzed by Western blot, and the mRNA levels of *p16^INK4A^*, *p21*, *IL‐1β*, *TNF‐α*, *IL‐6*, and *TGFβ1* quantified by quantitative polymerase chain reaction (qPCR) in IS‐treated *Cre^−^AhR^fl/fl^* mice, was all attenuated in IS‐treated *Cre^+^AhR^fl/fl^* mice (Figure 3c,d). These results suggested that the elevation in uremic toxin IS was able to induce renal injury and accelerate renal senescence. Knockout of tubular *AhR* blunted the IS‐induced renal injury, senescence, and fibrosis.

### 2.4 AhR Promoted IS‐Induced Cell Senescence and ECM Production in mTECs or Primary TECs

**Figure 4:**
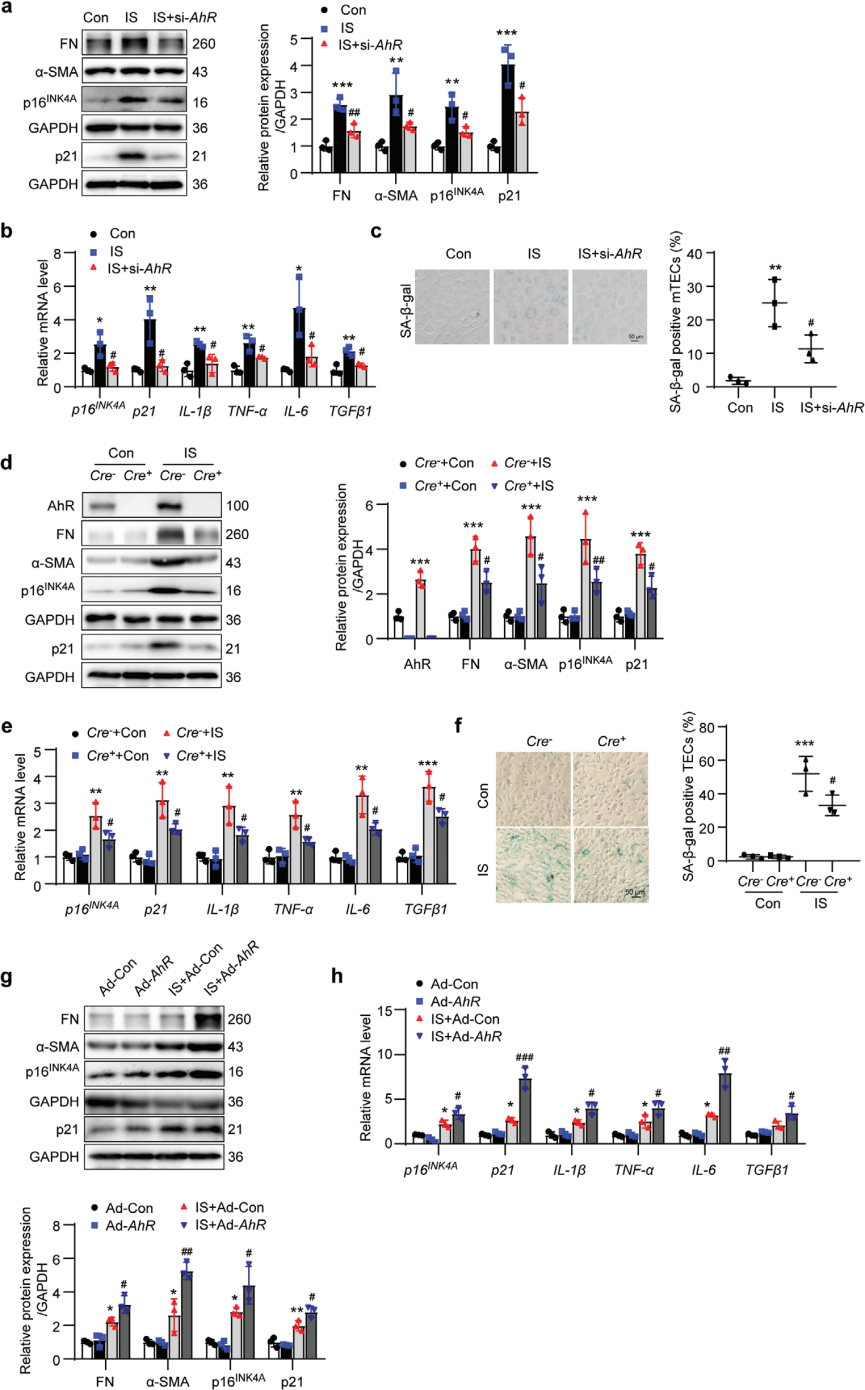
AhR promoted IS‐induced cell senescence and ECM production in mTECs or primary TECs. The mTECs were transfected with *AhR* siRNA (si‐*AhR*) for 12 h and then treated with IS (1000 µmol L^−1^) for an additional 36 h. a) Western blot images and quantitative data of FN, α‐SMA, p16^INK4A^, and p21 in mTECs. ^**^ *P* < 0.01, ^***^ *P* < 0.001 versus Con group, ^#^ *P* < 0.05, ^##^ *P* < 0.01 versus IS group (*n *= 3). b) The relative mRNA abundance of *p16^INK4A^*, *p21*, *IL‐1β*, *TNF‐α*, *IL‐6*, and *TGFβ1* in mTECs. ^*^ *P* < 0.05, ^**^ *P* < 0.01 versus Con group, ^#^ *P* < 0.05 versus IS group (*n *= 3). c) The SA‐β‐gal staining micrographs and quantitative data showing mTEC senescence. Scale bar, 50 µm. ^**^ *P* < 0.01 versus Con group, ^#^ *P* < 0.05 versus IS group (*n *= 3). Primary TECs isolated from *Cre^−^AhR^fl/fl^* mice (*Cre^−^*) or *Cre^+^AhR^fl/fl^* mice (*Cre^+^*) were treated with IS (1000 µmol L^−1^) for 36 h. d) Western blot images and quantitative data of AhR, FN, α‐SMA, p16^INK4A^, and p21 in primary TECs. ^***^ *P* < 0.001 versus *Cre^−^* +Con group, ^#^ *P* < 0.05, ^##^ *P* < 0.01 versus *Cre^−^* +IS group (*n *= 3). e) The relative mRNA abundance of *p16^INK4A^*, *p21*, *IL‐1β*, *TNF‐α*, *IL‐6*, and *TGFβ1* in primary TECs. ^**^ *P* < 0.01, ^***^ *P* < 0.001 versus *Cre^−^* +Con group, ^#^ *P* < 0.05 versus *Cre^−^* +IS group (*n *= 3). f) The SA‐β‐gal staining micrographs and quantitative data showing TEC senescence. Scale bar, 50 µm. ^***^ *P* < 0.001 versus *Cre^−^* +Con group, ^#^ *P* < 0.05 versus *Cre^−^* +IS group (*n *= 3). The mTECs were treated with IS (1000 µmol L^−1^) or adenovirus harboring *AhR* gene (Ad‐*AhR*) for 36 h. g) Western blot images and quantitative analysis of FN, α‐SMA, p16^INK4A^, and p21 expressions in mTECs. ^*^ *P* < 0.05, ^**^ *P* < 0.01 versus Ad‐Con group, ^#^ *P* < 0.05, ^##^ *P* < 0.01 versus IS+Ad‐Con group (*n *= 3). h) The relative mRNA abundance of cell senescence markers *p16^INK4A^* and *p21* as well as secretory factors *IL‐1β*, *TNF‐α*, *IL‐6*, and *TGFβ1* in mTECs. ^*^ *P* < 0.05 versus Ad‐Con group, ^#^ *P* < 0.05, ^##^ *P* < 0.01, ^###^ *P* < 0.001 versus IS+Ad‐Con group (*n *= 3). Data were shown as mean ± SD. Statistical analysis was performed by one‐way ANOVA with Tukey's multiple comparisons test (a‐c) and two‐way ANOVA with Tukey's multiple comparisons test (d‐h).

To confirm the effect of AhR on tubular cell senescence and ECM production in vivo, the expression of *AhR* was knocked down using *AhR*‐targeting siRNA (si‐*AhR*). The IS‐induced increase in AhR expression was abolished by transfection with si‐*AhR* (Figure S3, Supporting Information). Knockdown on *AhR* significantly alleviated IS‐induced increases in the protein levels of FN, α‐SMA, p16^INK4A^, and p21 (**Figure** **4**a). Knockdown on *AhR* also inhibited IS‐induced increases in the mRNA levels of *p16^INK4A^*, *p21*, *IL‐1β*, *TNF‐α*, *IL‐6*, and *TGFβ1* (Figure 4b). Knockdown on *AhR* also suppressed IS‐induced increase in SA‐β‐gal‐positive cells (Figure 4c). To further confirm the effect of AhR, cultured primary TECs from *Cre^−^AhR^fl/fl^ mice* (*Cre^−^*) and *Cre^+^AhR^fl/fl^* mice (*Cre^+^*) were treated with IS (Figure S4, Supporting Information). Consistently, the knockout of *AhR* significantly alleviated the IS‐induced increases in ECM production, cell senescence (Figure 4d,e), and SA‐β‐gal‐positive cells (Figure 4f).

Besides, the role of AhR was identified by transfection using recombinant adenovirus harboring *AhR* gene (Ad‐*AhR*) in cultured mTECs. Western blot results showed that the AhR level was significantly elevated in cells transfected with Ad‐*AhR* or treated with IS when compared with that in Ad‐Con. Moreover, the AhR level in the IS+Ad‐*AhR* group was higher than that in the IS+Ad‐Con group, which suggested that AhR was overexpressed in mTECs and IS was able to stimulate the expression of AhR (Figure S5, Supporting Information). Western blot results showed that compared with Ad‐Con cells, FN, α‐SMA, p16^INK4A^, and p21 expressions did not differ significantly in Ad‐*AhR* cells but were increased in IS+Ad‐Con cells. The levels of FN, α‐SMA, p16^INK4A^, and p21 were even higher in IS+Ad‐AhR cells (Figure 4g). Similar changes were observed in the mRNA levels of *p16^INK4A^*, *p21*, *IL‐1β*, *TNF‐α*, *IL‐6*, and *TGFβ1* (Figure 4h). These results indicated that the elevation of AhR following IS treatment promoted cell senescence and ECM production.

CH‐223191, as already reported previously, is a AhR antagonist and competitive AhR ligand.^[^ ^24^, ^25^ ^]^ As indicated by the protein levels of FN, α‐SMA, p16^INK4A^, and p21 as well as the mRNA levels of *p16^INK4A^*, *p21*, *IL‐1β*, *TNF‐α*, *IL‐6*, and *TGFβ1*, and SA‐β‐gal‐positive cells, the inhibition of AhR activation could significantly alleviate IS‐induced cell senescence and ECM production in mTECs (Figure S6a–c, Supporting Information) and human proximal tubular epithelial cells (HK‐2; Figure S6d–f, Supporting Information).

### 2.5 TEC‐Specific AhR Knockout Attenuated the Suppression of Mitochondrial Biogenesis in IR‐ or IS‐Treated Mice

**Figure 5:**
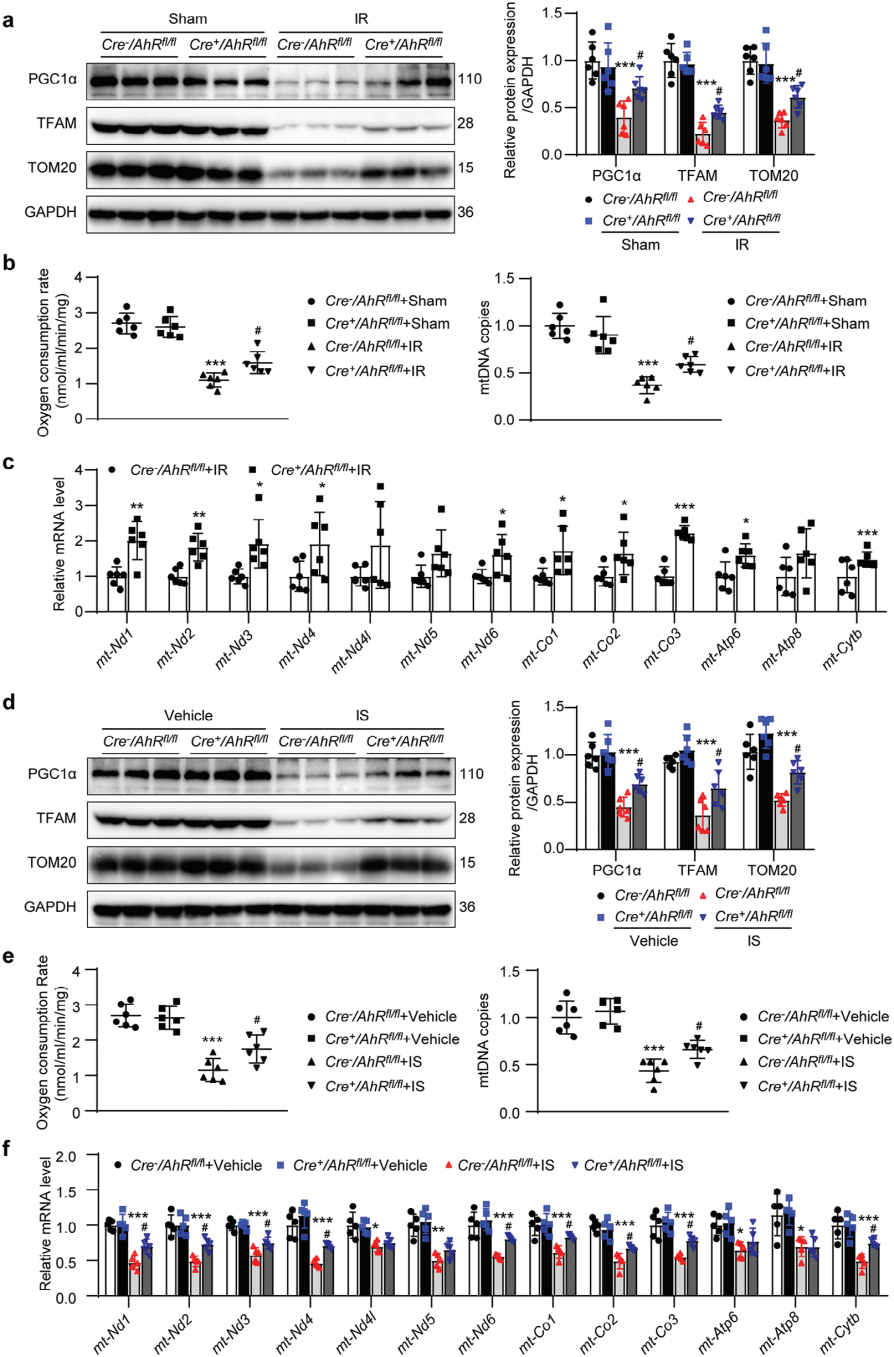
Renal tubular epithelial cell knockout of *AhR* blunted the suppression of mitochondrial biogenesis in the kidneys of IR‐ or IS‐treated mice. a) Western blot images and quantitative data of the protein levels of PGC1α, TFAM, and TOM20 in the kidneys of mice with or without IR surgery. ^***^ *P* < 0.001 versus *Cre^−^AhR^fl/fl^* +sham group, ^#^ *P* < 0.05 versus *Cre^−^AhR^fl/fl^* +IR group (*n *= 6). b) The renal oxygen consumption rate (OCR) and mitochondrial DNA (mtDNA) copies of mice with or without IR surgery. ^***^ *P* < 0.001 versus *Cre^−^AhR^fl/fl^* +sham group, ^#^ *P* < 0.05 versus *Cre^−^AhR^fl/fl^* +IR group (*n *= 6). c) qPCR showing the mRNA levels of 13 mtDNA in the kidneys of mice with or without IR surgery. ^*^ *P* < 0.05, ^**^ *P* < 0.01, ^***^ *P* < 0.001 versus *Cre^−^AhR^fl/fl^* +IR group (*n *= 6). d) Western blot images and quantitative data of the protein levels of PGC1α, TFAM and TOM20 in the kidneys of mice with or without IS treatment. ^***^ *P* < 0.001 versus *Cre^−^AhR^fl/fl^* +vehicle group, ^#^ *P* < 0.05 versus *Cre^−^AhR^fl/fl^* +IS group (*n *= 6). e) Renal OCR and mtDNA of mice with or without IS treatment. ^***^ *P* < 0.001 versus *Cre^−^AhR^fl/fl^* +vehicle group, ^#^ *P* < 0.05 versus *Cre^−^AhR^fl/fl^* +IS group (*n *= 6). f) qPCR showing the mRNA levels of 13 mtDNA in the kidneys of mice with or without IS treatment. ^*^ *P* < 0.05, ^**^ *P* < 0.01, ^***^ *P* < 0.001 versus *Cre^−^AhR^fl/fl^* +vehicle group, ^#^ *P* < 0.05 versus *Cre^−^AhR^fl/fl^* +IS group (*n *= 5). Data were shown as mean ± SD. Statistical analysis was performed by two‐tailed unpaired Student's *t*‐test (*mt‐Nd1*, *mt‐Nd2*, *mt‐Nd4*, *mt‐Nd5*, *mt‐Co2*, *mt‐Co3*, *Atp6*, *Atp8*, *mt‐Cytb* of c), two‐tailed unpaired Welch’ *t*‐test (*mt‐Nd3*, *mt‐Nd4l*, *mt‐Nd6*, *mt‐Co1* of c), and two‐way ANOVA with Tukey's multiple comparisons test (a, b, d, e, f).

Insufficient mitochondrial biogenesis induces cell senescence. Therefore, we explored whether AhR regulates mitochondrial biogenesis. PGC1α is a key regulator of mitochondrial biogenesis and, as a transcriptional coactivator, interacts with nuclear respiratory factors 1 and 2 to regulate the transcription of mitochondrial transcription factor A (*TFAM*), and thereby determine the mitochondrial biogenesis and cellular mitochondrial content.^[^ ^26^ ^]^ Western blot results showed that PGC1α, TFAM, and TOM20, which are markers of cellular mitochondrial biogenesis, were significantly decreased in *Cre^−^AhR^fl/fl^* mice following IR surgery, and the suppression of PGC1α, TFAM, and translocase of outer mitochondrial membrane 20 (TOM20 expressions was blunted in *Cre^+^AhR^fl/fl^* mice (**Figure** **5**a). Consistent with these results, the renal oxygen consumption rate (OCR) and mitochondrial DNA (mtDNA) copies were significantly decreased in *Cre^−^AhR^fl/fl^* mice following IR surgery, and these reductions were blunted in *Cre^+^AhR^fl/fl^* mice (Figure 5b). Furthermore, qPCR results showed that the mRNA levels of 13 mtDNA in IR‐treated *Cre^+^AhR^fl/fl^* mice were higher than those in IR‐treated *Cre^−^AhR^fl/fl^* mice (Figure 5c). These results suggested that mitochondrial biogenesis was suppressed in IR mice, which was alleviated by tubular‐specific knockout of *AhR*. Furthermore, mitochondrial biogenesis was observed in IS‐treated mice. Similarly, IS administration suppressed mitochondrial biogenesis, and tubular‐specific knockout of *AhR* attenuated IS‐induced the inhibition (Figure 5d–f). These data suggested that the accumulation of uremic toxins suppressed mitochondrial biogenesis via upregulation of AhR.

### 2.6 AhR Promoted IS‐Induced the Suppression of Mitochondrial Biogenesis in mTECs or Primary TECs

**Figure 6:**
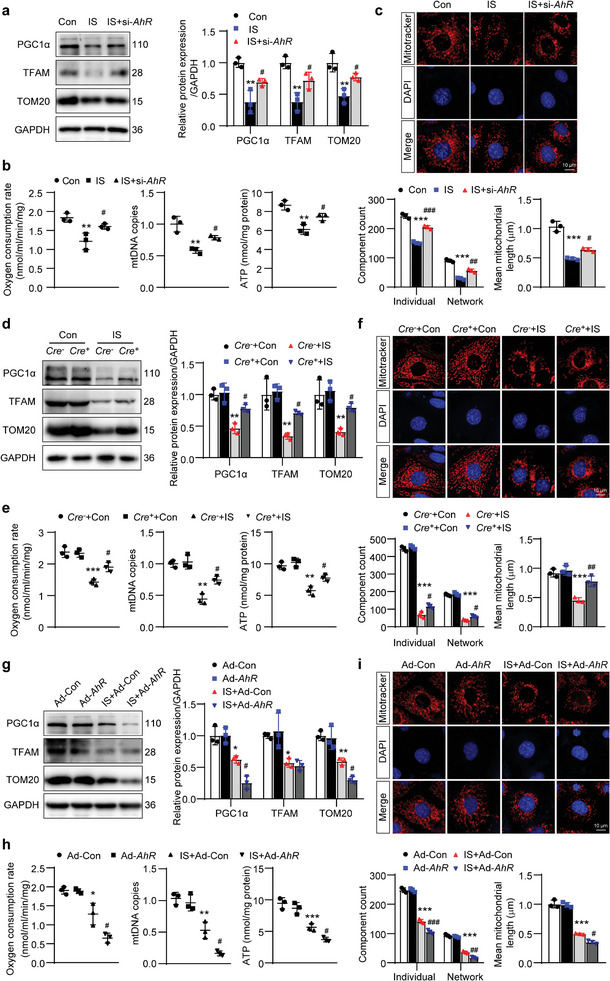
AhR promoted IS‐induced the suppression of mitochondrial biogenesis in mTECs or primary TECs. a) Western blot images and quantitative data of PGC1α, TFAM and TOM20 in mTECs. ^**^ *P* < 0.01 versus Con group, ^#^ *P* < 0.05 versus IS group (*n *= 3). b) The measurement of OCR, mtDNA copies, and the ATP content in mTECs. ^**^ *P* < 0.01 versus Con group, ^#^ *P* < 0.05 versus IS group (*n *= 3). c) Representative images of Mitotracker staining in mTECs. Scale bar, 10 µm. MiNa‐based quantification of the mitochondrial morphological parameters, including individual mitochondria, mitochondrial networks, and mitochondrial length. ^***^ *P* < 0.001 versus Con group, ^#^ *P* < 0.05, ^##^ *P* < 0.01, ^###^ *P* < 0.001 versus IS group (*n *= 3). d) Western blot images and quantitative data of PGC1α, TFAM and TOM20 in primary TECs. ^**^ *P* < 0.01 versus *Cre^−^* +Con group, ^#^ *P* < 0.05 versus *Cre^−^* +IS group (*n *= 3). e) The measurement of OCR, mtDNA copies and the ATP content in primary TECs. ^**^ *P* < 0.01, ^***^ *P* < 0.001 versus *Cre^−^* +Con group, ^#^ *P* < 0.05 versus *Cre^−^* +IS group (*n *= 3). f) Representative images of Mitotracker staining in primary TECs. Scale bar, 10 µm. MiNa‐based quantification of the mitochondrial morphological parameters, including individual mitochondria, mitochondrial networks, and mitochondrial length. ^***^ *P* < 0.001 versus *Cre^−^* +Con group, ^#^ *P* < 0.05, ^##^ *P* < 0.01 versus *Cre^−^* +IS group (*n *= 3). g) Western blot images and quantitative analysis of PGC1α, TFAM and TOM20 expressions in mTECs. ^*^ *P* < 0.05, ^**^ *P* < 0.01 versus Ad‐Con group, ^#^ *P* < 0.05 versus IS+Ad‐Con group (*n *= 3). h) The measurement of OCR, mtDNA copies and the ATP content in mTECs. ^*^ *P* < 0.05, ^**^ *P* < 0.01, ^***^ *P* < 0.001 versus Ad‐Con group, ^#^ *P* < 0.05 versus IS+Ad‐Con group (*n *= 3). i) Representative images of Mitotracker staining in mTECs. Scale bar, 10 µm. MiNa‐based quantification of the mitochondrial morphological parameters, including individual mitochondria, mitochondrial networks, and mitochondrial length. ^***^ *P* < 0.001 versus Ad‐Con group, ^#^ *P* < 0.05, ^##^ *P* < 0.01, ^###^ *P* < 0.001 versus IS+Ad‐Con group (*n *= 3). Data were shown as mean ± SD. Statistical analysis was performed by one‐way ANOVA with Tukey's multiple comparisons test (a‐c) and two‐way ANOVA with Tukey's multiple comparisons test (d‐i).

The role of AhR in mitochondrial biogenesis was observed in vitro. IS‐induced reductions in PGC1α, TFAM, and TOM20 expressions were alleviated in mTECs transfected with si‐*AhR* or in primary TECs isolated from *Cre^+^AhR^fl/fl^* mice (**Figure** **6**a,d). Similar changes were observed in the OCR, mtDNA copies and cellular ATP content (Figure 6b,e). To assess mitochondrial morphology, immunofluorescence staining with the mitochondria‐specific fluorescent dye Mitotracker was performed in mTECs or primary TECs. The mitochondrial morphological changes were analyzed using Mitochondrial Network Analysis (MiNa), a semi‐automated ImageJ plugin that enabled the quantification of network parameters. The results revealed a significant reduction in individual mitochondria, mitochondrial networks, and the mean mitochondrial length in IS‐treated cells when compared with Con cells, suggesting that IS reduced mitochondrial mass and induced fragmentation. Knockdown on *AhR* in mTECs (Figure 6c) and knockout of *AhR* in primary TECs (Figure 6f) alleviated this effect. On the contrary, compared with Ad‐Con cells, no obvious change in PGC1α, TFAM and TOM20 expressions, as well as OCR, mtDNA copies, ATP content, and Mitotracker signals, were observed in Ad‐*AhR* cells whereas all these parameters were decreased in IS+Ad‐Con cells. Overexpression of *AhR* aggravated these changes (Figure 6g–i). The effects of IS and overexpression of *AhR* on mitochondrial biogenesis were also confirmed by observing mRNA levels of 13 mtDNA by qPCR (Figure S7, Supporting Information). These results suggested that knockdown or knockout of *AhR* alleviated IS‐induced mitochondrial biogenesis impairment while overexpression of *AhR* enhanced the effect of IS.

### 2.7 Overexpression of PGC1α Alleviated IS‐Induced Cell Senescence and ECM Production

**Figure 7:**
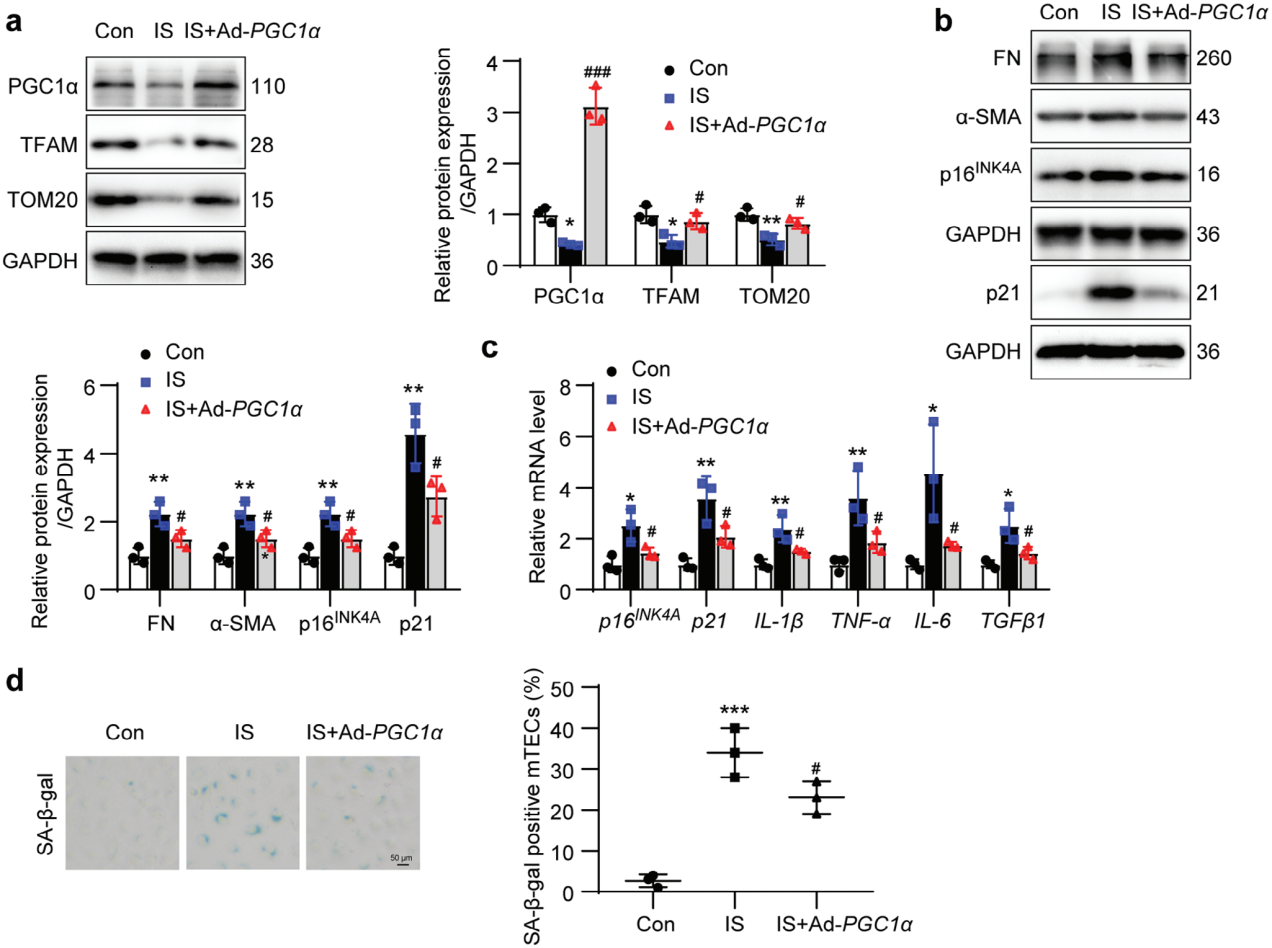
Overexpression of *PGC1α* alleviated IS‐induced cell senescence and ECM production. The mTECs were treated with IS (1000 µmol L^−1^) or adenovirus harboring *PGC1α* gene (Ad‐*PGC1α*) for 36 h. a) Western blot images and quantitative analysis showing the expressions of PGC1α, TFAM and TOM20 in mTECs. ^*^ *P* < 0.05, ^**^ *P* < 0.01 versus Con group, ^#^ *P* < 0.05, ^###^ *P* < 0.001 versus IS group (*n *= 3). b) Western blot images and quantitative analysis showing the expressions of FN, α‐SMA, p16^INK4A^ and p21 in mTECs. ^**^ *P* < 0.01 versus Con group, ^#^ *P* < 0.05 versus IS group (*n *= 3). c) qPCR analysis of the relative mRNA levels of *p16^INK4A^*, *p21*, *IL‐1β*, *TNF‐α*, *IL‐6*, and *TGFβ1* in mTECs. ^*^ *P* < 0.05, ^**^ *P* < 0.01 versus Con group, ^#^ *P* < 0.05 versus IS group (*n *= 3). d) The SA‐β‐gal staining micrographs and quantitative analysis showing the mTEC senescence. Scale bar, 50 µm. ^***^ *P* < 0.001 versus Con group, ^#^ *P* < 0.05 versus IS group (*n *= 3). Data were shown as mean ± SD. Statistical analysis was performed by one‐way ANOVA with Tukey's multiple comparisons test (a‐d).

To identify the role of PGC1α‐mediated mitochondrial biogenesis in cell senescence, PGC1α was overexpressed by transfection of recombinant adenovirus harboring *PGC1α* gene (Ad‐*PGC1α*) in mTECs. Overexpression of *PGC1α* reversed the IS‐induced reductions in TFAM and TOM20 protein levels (**Figure** **7**a). Overexpression of *PGC1α* also inhibited IS‐induced increases in FN, α‐SMA, p16^INK4A^, and p21 protein levels (Figure 7b), and *p16^INK4A^*, *p21*, *IL‐1β*, *TNF‐α*, *IL‐6*, and *TGFβ1* mRNA levels (Figure 7c). IS‐induced increase in SA‐β‐gal‐positive signals was also suppressed by *PGC1α* overexpression (Figure 7d). These results indicated that the decrease in PGC1α‐mediated mitochondrial biogenesis was implicated in IS‐induced cell senescence and ECM production.

### 2.8 AhR Promoted PGC1α Degradation via Ubiquitin Modification

**Figure 8:**
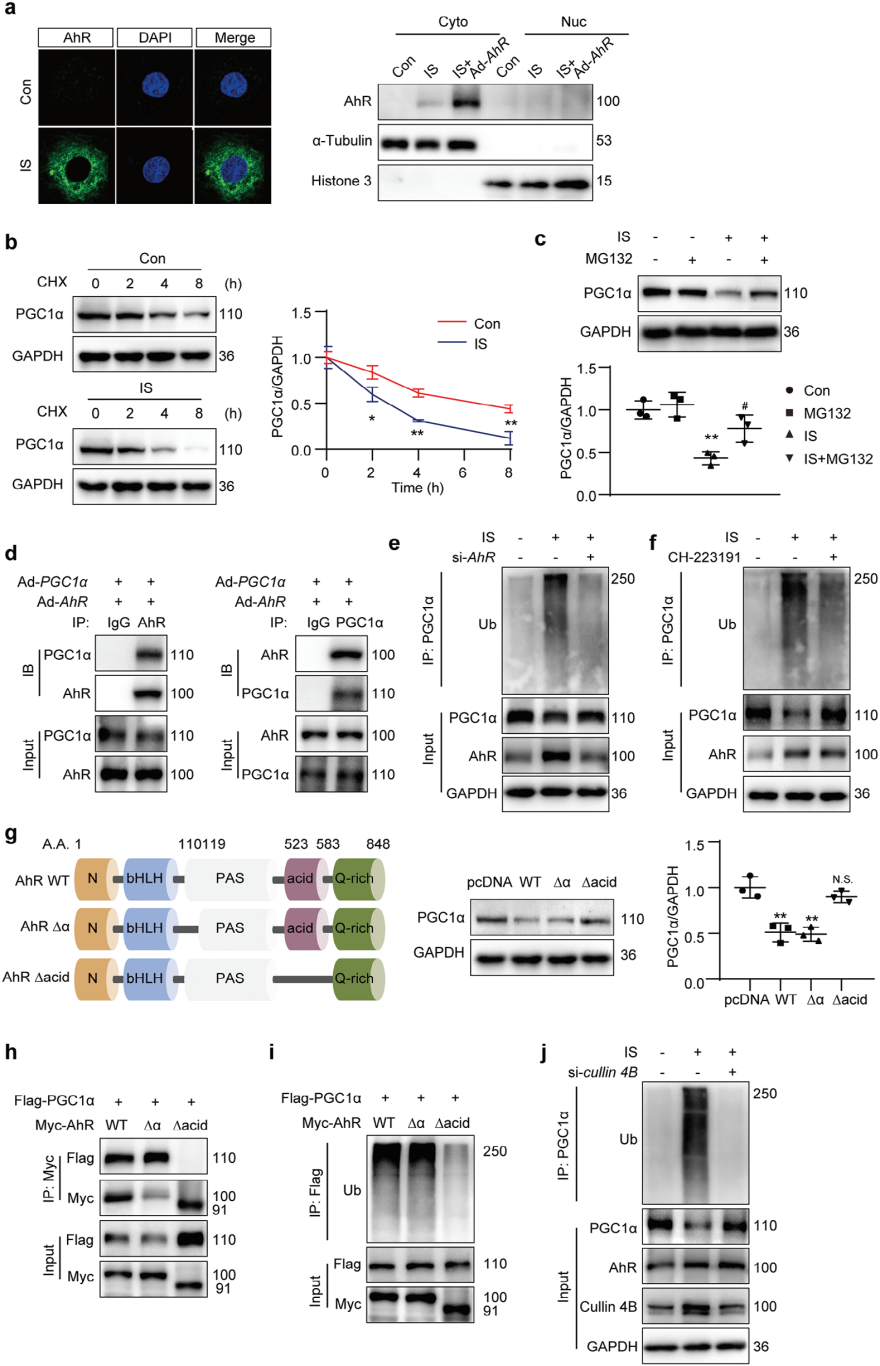
AhR promoted PGC1α degradation via ubiquitin modification. a) Immunofluorescence micrographs and Western blot presenting AhR distribution in mTECs after IS treatment (1000 µmol L^−1^) for 12 h, with or without transfection using adenovirus carrying AhR gene (Ad‐*AhR*) for 36 h. b) Western blot images and quantitative data detecting the PGC1α in mTECs with IS treatment (1000 µmol L^−1^) for 36 h. Cells were treated with cycloheximide (CHX, 10 µmol L^−1^) for the indicated durations before being harvested. ^*^ *P* < 0.05, ^**^ *P* < 0.01 versus Con group (*n *= 3). c) Western blot images and quantitative data detecting the PGC1α in mTECs subjected to 36‐h IS treatment (1000 µmol L^−1^) in the presence of MG132 (20 µmol L^−1^) for 4 h. ^**^ *P* < 0.01 versus Con group, ^#^ *P* < 0.05 versus IS group (*n *= 3). d) Co‐IP of AhR and PGC1α in mTECs cotransfected with Ad‐*AhR* and Ad‐*PGC1α* with IS stimulation (1000 µmol L^−1^) for 48 h. The lysates were immunoprecipitated with control IgG, anti‐AhR, or anti‐PGC1α antibodies, followed by Western blot with the indicated antibody (*n *= 3). e,f) IP analyzing the ubiquitination of PGC1α in mTECs. The cells were transfected with *AhR* siRNA (si‐*AhR*; e) or treated with AhR inhibitor CH‐223191 (10 µmol L^−1^; f) in the presence of IS (1000 µmol L^−1^) for 48 h. MG132 (20 µmol L^−1^) was added to the medium 4 h before cell harvest. The lysates were immunoprecipitated with anti‐PGC1α antibody, followed by Western blot with anti‐ubiquitin (Ub) antibody (*n *= 3). g) Schematic of full‐length and truncated AhR mutants. Western blot images and quantitative data detecting the PGC1α in mTECs. The cells were transfected with Myc‐tagged pcDNA, AhR full‐length (WT), 110–119 amino acid‐deletion mutant (Δα), or acidic domain‐deletion mutant (Δacid) vectors with IS stimulation (1000 µmol L^−1^) for 48 h. ^**^ *P* < 0.01, N.S. (no significant difference) versus pcDNA group (*n *= 3). h) Co‐IP of AhR or mutants with PGC1α in HEK293T cells cotransfected with plasmids encoding Flag‐tagged PGC1α and Myc‐tagged AhR WT, AhR Δα or AhR Δacid with IS stimulation (1000 µmol L^−1^) for 48 h. The lysates were immunoprecipitated with anti‐Myc antibody, followed by Western blot with anti‐Flag antibody (*n *= 3). i) IP of AhR or mutants with PGC1α in HEK293T cells cotransfected with plasmids encoding Flag‐tagged PGC1α and Myc‐tagged AhR WT, AhR Δα or AhR Δacid in the presence of IS (1000 µmol L^−1^) for 48 h. MG132 (20 µmol L^−1^) was added to the medium 4 h before cell harvest. The lysates were immunoprecipitated with anti‐Flag antibody, followed by Western blot with anti‐Ub antibody (*n *= 3). j) IP analysis of PGC1α ubiquitination in mTECs transfected with *cullin 4B* siRNA (si‐*cullin 4B*) in the presence of IS (1000 µmol L^−1^) for 48 h. MG132 (20 µmol L^−1^) was added to the medium 4 h before cell harvest. The lysates were immunoprecipitated with anti‐PGC1α antibody, followed by Western blot with anti‐ubiquitin (Ub) antibody (*n *= 3). Data were shown as mean ± SD. Statistical analysis was performed by two‐way ANOVA with Tukey's multiple comparisons test (b, c, g).

AhR has been traditionally recognized as a nuclear transcriptional factor, but was recently identified as possessing E3 ubiquitin ligase activity. To explore the exact mechanism of AhR‐mediated inhibition of PGC1α expression, the distribution of AhR was measured upon IS stimulation. Immunofluorescence analysis showed that IS stimulated the expression of AhR and AhR was mainly located in the cytoplasm, which was also confirmed in the kidneys of IR and IS mice. To further identify the subcellular distribution of AhR, the cytoplasmic and nuclear fractions were separated. The results showed that increased AhR following IS stimulation was only observed in the cytoplasm but not in the nucleus (**Figures** **8**a and 1k). Intriguingly, the mRNA level of *PGC1α* was unaffected in the IR‐ or IS‐treated mouse kidneys (Figure S8a, Supporting Information) and in the IS‐stimulated mTECs with knockdown or overexpression of *AhR* (Figure S8b, Supporting Information). In contrast, the protein level of PGC1α was significantly reduced (Figures 5a,d and 6a). To clarify the mechanism by which AhR repressed the protein level of PGC1α, cycloheximide (CHX) was used to block the protein synthesis. The result showed that IS treatment accelerated the degradation of the PGC1α protein (Figure 8b). Application of MG132, a proteasome inhibitor, attenuated the decrease in PGC1α induced by IS, which suggested that the enhanced PGC1α degradation was mediated via ubiquitination and the proteasome pathway (Figure 8c). To identify the interaction between AhR and PGC1α, adenovirus harboring *AhR* and *PGC1α* were cotransfected into mTECs. The co‐immunoprecipitation (Co‐IP) result showed that the anti‐AhR antibody was able to immunoprecipitate PGC1α and the anti‐PGC1α antibody could also immunoprecipitate AhR (Figure 8d). The interaction of endogenous AhR and PGC1α was observed following IS stimulation (Figure S8c, Supporting Information). Accordingly, IS increased the ubiquitin modification of PGC1α (Figure S8d, Supporting Information), while knockdown on *AhR* abolished this increase (Figure 8e). In addition, the AhR inhibitor CH‐223191 decreased the ubiquitination of PGC1α induced by IS in both mTECs (Figure 8f) and HK‐2 (Figure S8e, Supporting Information).

The functional domains of AhR have been well documented in the previous study. Deletion of amino acid residues 110–119 abolishes the nuclear translocational ability of AhR, while deletion of the acidic domain (523–583 amino acids) abolishes the ubiquitin ligase activity of AhR. To further clarify the exact mechanism of AhR in regulating the protein level of PGC1α, several pcDNA vectors encoding Myc‐tagged AhR WT (carrying the full‐length sequence) and two truncated AhR mutants, AhR Δα (deletion of 110–119 amino acid residues) and AhR Δacid (deletion of the acidic domain), were constructed and transferred into mTECs.^[^ ^21^, ^27^ ^]^ AhR WT and AhR Δα decreased the protein level of PGC1α when compared with that of pcDNA, while AhR Δacid reversed this change (Figure 8g). The Co‐IP assays demonstrated that PGC1α co‐precipitated with AhR WT and AhR Δα, but not AhR Δacid, indicating that the AhR acidic domain was necessary for AhR to interact with PGC1α (Figure 8h). Compared with cells transfected with AhR WT or AhR Δα, the ubiquitin modification of PGC1α was decreased in cells transfected with AhR Δacid (Figure 8i).

Cullin 4B is the enzymatic component of the ubiquitin ligase complex. To further identify that AhR decreased PGC1α by cullin 4B‐based E3 ubiquitin ligase and proteasomal degradation, *cullin 4B* was knockdown by siRNA (si‐*cullin 4B*). As expected, knockdown on *cullin 4B* by siRNA alleviated the IS‐induced increase in PGC1α ubiquitination (Figure 8j). Besides, knockdown on *cullin 4B* abolished the IS‐induced decrease in the PGC1α protein level (Figure S8f,g, Supporting Information). The Co‐IP results showed that both the anti‐Myc and anti‐Flag antibodies were able to immunoprecipitate cullin 4B by exogenously overexpressing Myc‐tagged AhR WT or Flag‐tagged PGC1α (Figure S8h,i, Supporting Information). Endogenous interaction between AhR and cullin 4B was also confirmed to be enhanced by IS (Figure S8j, Supporting Information). Those data indicated that AhR decreased PGC1α by the E3 ubiquitin ligase activity but not nuclear transcriptional factor activity.

## 3 Discussion

The accumulation of uremic toxins in the bodily fluids is harmful to all tissues and organs, but the exact mechanism has long been ignored. Recently, AhR was accepted as a receptor for uremic toxins. As a result, the effects of uremic toxin‐activated AhR are of great interest to many researchers. Recent evidence has shown that the uremic toxin IS‐activated AhR promotes thrombosis and endovascular injury by inhibiting tissue factor ubiquitination and degradation.^[^ ^28^ ^]^ However, understanding the pathological function of AhR in intrinsic renal cells in CKD is quite limited. In the study, our data suggested that AhR was increased in CKD murine models following the accumulation of uremic toxins in bodily fluids and was mainly distributed in mitochondria‐rich tubular epithelial cells. Tubular‐specific knockout of *AhR* mitigated cell senescence and renal fibrosis. Mechanically, AhR was implicated in the suppression of mitochondrial biogenesis by increasing PGC1α ubiquitin degradation.

CKD is an irreversible health issue with high morbidity in the older population.^[^ ^29^ ^]^ Renal fibrosis has been recognized as a consequence of accelerated renal cell senescence under pathological conditions.^[^ ^8^ ^]^ Senescent cells exhibit a senescence‐associated secretory phenotype that produces various proinflammatory and profibrotic cytokines. These cytokines are well‐documented to facilitate renal fibrosis by acting on tubular cells and interstitial fibroblasts, and increasing inflammatory cell infiltration. Following the decline in renal function, the retention of uremic toxins in the bodily fluids facilitates renal dysfunction in CKD, and thus creates a vicious circle and accelerates the worsening of renal function. Until 2003, more than 90 uremic toxins were identified by the European Uremic Toxin Work Group.^[^ ^30^ ^]^ A recent study reported that ≈130 uremic toxins are increased in CKD mice and are correlated with a decline in GFR.^[^ ^31^ ^]^ Clarifying the exact mechanism of uremic toxins in the development of CKD is an essential issue in renal research. Recent studies have shown that the administration of the uremic toxin IS suppresses the expression of klotho and promotes cell senescence and renal fibrosis.^[^ ^13^, ^32^, ^33^, ^34^ ^]^ although the underlying mechanism has not been clarified. In this study, we found that AhR was significantly upregulated in mouse kidneys following the accumulation of uremic toxins in bodily fluids. Besides, the elevation of AhR was observed in IS‐treated cultured TECs. Previous studies have shown increased AhR activity in patients with end‐stage renal disease and in adenine‐induced CKD mice. In addition, AhR activity had a positive correlation with serum IS level.^[^ ^22^, ^23^, ^24^, ^25^, ^26^, ^27^, ^28^ ^]^ These results suggested that the accumulation of uremic toxins was able to initiate the activation of their endogenous receptor AhR. However, the mechanism of upregulated AhR induced by IS is unclear and was not within the scope of the current investigation.

The elevation of AhR was mainly observed in renal TECs. Renal TEC‐specific knockout of *AhR* attenuated renal functional deterioration and fibrosis, which suggested that the elevation of AhR was implicated in accelerating renal injury. Increases in senescent markers were also observed in IR‐ and IS‐treated mice, while knockout of renal tubular *AhR* slowed the development of renal senescence. These results suggested that an increase in AhR was implicated in accelerating renal senescence. A similar phenomenon has been observed in cardiac and liver studies, which showed that the elevation of AhR is associated with organ fibrosis and cell senescence.^[^ ^16^, ^17^, ^18^, ^19^, ^20^, ^21^, ^22^, ^23^, ^24^, ^25^, ^26^, ^27^, ^28^, ^29^, ^30^, ^31^, ^32^, ^33^, ^34^, ^35^ ^]^

Senescence is inevitably accompanied by a decline in metabolic rate, which is correlated with the production of ATP in the mitochondria. In the study, our results showed that OCR, mtDNA copies, cellular ATP content, and mitochondrial content were significantly decreased in IR‐ or IS‐treated mouse kidneys, and were blunted by knocking out *AhR* in TECs. These data suggested that the elevation of AhR was involved in the suppression of mitochondrial biogenesis.

The mitochondria are an intracellular organelle with high metabolic rates. Mitochondrial biogenesis is maintained through the continuous production of neonatal mitochondria to replace degenerated mitochondria. PGC1α is a key regulator of mitochondrial biogenesis.^[^ ^36^, ^37^ ^]^ In the experiment, PGC1α was significantly decreased in IR‐ or IS‐treated mouse kidneys and IS‐treated mTECs. Overexpression of *PGC1α* in IS‐treated cells restored mitochondrial biogenesis and attenuated the appearance of senescence. These results suggested that the suppression of mitochondrial biogenesis due to the downregulation of PGC1α was related to renal senescence.

Another interesting finding in the current study is that AhR was a negative regulator of PGC1α albeit without relying on its nuclear transcriptional activity. A previous study reported that AhR acts as a nuclear transcriptional factor that binds to the promoters of cyclooxygenase 2, *FN*, collagen IV and connective tissue growth factor, stimulates the expression of these genes, and thus initiates mesangial cell proliferation, renal glomerular fibrosis and macrophage infiltration in mice with diabetic nephropathy.^[^ ^38^ ^]^ To our surprise, AhR was hardly detected in the nucleus either by Western blot or immunofluorescence in our study. A novel activity of AhR was recently revealed that AhR can act as a ligand‐activated E3 ubiquitin ligase.^[^ ^21^, ^39^, ^40^ ^]^ Further investigation showed that the decrease in PGC1α was only observed at the protein level but not at the mRNA level. PGC1α can be modified by ubiquitination and interact with AhR. The AhR mutant lack of transcriptional activity did not influence PGC1α, while the mutant lack of E3 ubiquitin ligase abolished the ability to decrease PGC1α. All these results suggested that AhR decreased PGC1α via promoting ubiquitination and proteasomal degradation but not transcriptional regulation. The ligand‐activated AhR acts as an adaptor to bridge the cullin 4B E3 ubiquitin ligase complex and substrates.^[^ ^21^ ^]^ Knockdown on *cullin 4B* could alleviate the IS‐induced increase in the PGC1α ubiquitination. Similar studies showed that kynurenine‐activated AhR bridged the cullin 4B E3 ubiquitin ligase complex and substrates, stimulator of interferon response CGAMP interactor^[^ ^41^ ^]^ and runt‐related transcription factor 2^[^ ^42^ ^]^, facilitating their degradation via the ubiquitin‐proteasome pathway.

Although this study revealed that the elevation of AhR was implicated in renal senescence and functional deterioration, some questions remain unclear, such as how IS upregulates AhR, why AhR is retained in the cytoplasm rather than translocates into the nucleus to act as a transcriptional factor, and whether other uremic toxins have similar effects after binding to AhR.

In conclusion, our data demonstrated that the uremic toxin receptor AhR was elevated following the retention of uremic toxins in the bodily fluids. As a novel ligand‐activated E3 ubiquitin ligase, AhR promoted the ubiquitination and proteasomal degradation of PGC1α, which suppressed mitochondrial biogenesis and thereby accelerated renal senescence and fibrosis. Our study highlights that the elimination of retained uremic toxins to avoid AhR upregulation is beneficial in patients with CKD. The results of this study may provide an insight into uremic toxin‐induced damage to other organs such as the heart and brain.

## 4 Experimental Section

### Generation of TEC‐Specific AhR Knockout Mice

For the generation of *AhR^flox/+^* inbred mouse line, the targeting vector was inserted into two loxP sites on the flank of the fifth and seventh exons of AhR in the zygotes of C57BL/6N mice. *AhR^flox/+^* mice were generated using Cyagen (Suzhou, China). *AhR^flox/+^* mice were crossed with *Ggt1‐Cre* mice (expressing Cre under the control of *Ggt1* promoter; Jackson, https://www.jax.org/strain/012841) to generate TEC‐specific AhR knockout mice (*Cre^+^AhR^fl/fl^*). *AhR^fl/fl^* mice without Cre littermates were served as controls (*Cre^−^AhR^fl/fl^*). The mice were genotyped by PCR amplification of genomic DNA isolated from the tail tissue. The primers used for genotyping are listed in Table S1 (Supporting Information).

### Renal Histology and Immunohistochemical Staining

The mouse kidney samples were fixed in 10% formalin, embedded in paraffin, and sectioned into at 4‐µm‐thick slices. HE, PAS, Masson, and Sirius Red staining were performed after deparaffinization and rehydration. Histopathological changes of tubular injury, such as tubular dilation, disruption, loss of brush borders and cast formation, were evaluated in a blind manner, and scored as 0, 1, 2, 3, or 4 based on injury areas of 0%, <25%, 26%–50%, 51%–75%, and >76%, respectively, in 10 randomly chosen and non‐overlapping fields. Interstitial fibrosis was evaluated based on the proportion of areas that stained positively with Masson staining in six random fields for each mouse. The distribution of AhR was determined by immunohistochemical staining of mouse kidney slices using specific anti‐AhR antibody (1:200, MA1‐514, Thermo Fisher Scientific, Waltham, MA).

### Animal Preparation

Eight‐week‐old male mice (weight 20–25 g) were subjected to IR surgery. In brief, after anesthetized, the left kidney was exposed, and the renal pedicel was clamped for 40 min with non‐traumatic microvascular clamps. The body temperature was maintained at 37 °C during surgery. The right kidney was excised at 14 days after surgery. The sham‐operated animals were subjected to the same procedure, except for renal‐pedicle clamping and kidney excision. These mice were euthanized 28 days after IR surgery, and serum and kidney tissues were collected for further experiments.

For the preparation of IS‐treated animals, IS was dissolved in sterile water at a concentration of 25 mg mL^−1^. The mice were implemented right kidney excision. Three days after surgery, IS (200 mg kg^−1^ per day) was administered via oral gavage. The mice receiving the same volume of sterile water by gavage were used as controls. These mice were euthanized 50 days after IS gavage. The serum and kidney tissues were collected for further experiments.

### Cell Culture and Treatment

MTECs were obtained as a gift from Prof. Lan's lab, the Chinese University of Hong Kong, acquired initially from Dr. Jeffrey B. Kopp, NIH.^[^ ^43^ ^]^ HK‐2 cells were purchased from FuHeng Biology (FH0228). MTECs and HK‐2 were cultured in DMEM‐F12 (Hyclone, Logan, Utah) supplemented with 10% fetal bovine serum (ExCell Bio, Shanghai, China) and 1% (v v^−1^) penicillin–streptomycin (Gibco, Thermo Fisher Scientific, Waltham, MA) at 37 °C in a 5% CO_2_ incubator.

Primary TECs were isolated from *Cre^+^AhR^fl/fl^* or *Cre^−^AhR^fl/fl^* mice. The renal cortices were chopped and digested with 1 mg mL^−1^ collagenase II in phosphate‐buffered saline (PBS) at 37 °C for 10 min. DMEM‐F12 supplemented with 10% FBS was used to stop digestion. The mixture was passed sequentially through 100 and 70 µm filters (BD Falcon). The filtrate was collected by the bottom and centrifugated. The cells were seeded in a culture dish in DMEM‐F12 supplemented with 10% FBS and 1% (v v^−1^) penicillin–streptomycin.

IS (I3875, Sigma‐Aldrich, Darmstadt, Germany) was dissolved in sterile water at a concentration of 250 mmol L^−1^. MTECs were treated with IS by incubating at the indicated concentration of IS for the indicated durations. MTECs or HK‐2 were treated with CH‐223191 (C8124, Sigma‐Aldrich, Darmstadt, Germany; 10 µmol L^−1^) for 36 h. MTECs were treated with MG132 (S1748, Beyotime, Shanghai, China; 20 µmol L^−1^) for 4 h or CHX (HY‐12320, MCE, Shanghai, China; 10 µmol L^−1^) for the indicated durations.

### Transfection and Plasmids Construction

Knockdown on *AhR* in mTECs was performed by transfecting *AhR* siRNA (si‐AhR, 50 nMmol/L) using the RNAiMAX reagent according to the manufacturer's instructions (13 778 030, Thermo Fisher Scientific, Waltham, MA). Mouse si‐*AhR* (5′‐UCCCACAUCCGCAUGAUUA‐3′) and si‐*cullin 4B* (5′‐GCUGAAUUUAAAGAGGGCAAA‐3′) were designed and synthesized by Biotend (Shanghai, China). Full‐length and truncated mouse AhR fragments were established using PCR procedure. The products were cloned into Myc‐tagged pcDNA3.1 vectors. The plasmids were delivered into mTECs using the Lipofectamine 3000 reagent (L3000008, Thermo Fisher Scientific, Waltham, MA). Overexpression of *AhR* or *PGC1α* in mTECs was performed by the transfection for 36 h with Ad‐*AhR* (containing Flag tag) or Ad‐*PGC1α* (containing Flag tag), respectively.

### RNA Extraction and qPCR

RNA was extracted from renal tissues or cultured cells using Trizol (Biocolor Bioscience, Shanghai, China). Then, cDNA was synthesized by reverse transcription PCR using the PrimeScript RT reagent kit (Takara, Japan). Subsequently, qPCR was performed using the SYBR Green Master Mix (Toyobo, Japan) on an Applied Biosystems 7300 Plus system. The relative mRNA levels were normalized to glyceraldehyde‐3‐phosphate dehydrogenase (*GAPDH*). The primers used for amplification are listed in Table S2 (Supporting Information).

### Western Blot

Renal tissues and cultured cells were lysed in a 2% sodium dodecyl sulphate (SDS) lysis buffer. After centrifugation, the supernatant containing the proteins was mixed with loading buffer and separated on SDS‐polyacrylamide gels. Subsequently, proteins were electrophoretically transferred onto a polyvinylidene fluoride membrane (Millipore, Darmstadt, Germany). Then, the membrane was blocked and incubated with primary antibodies against: AhR (1:2000, MA1‐514, Thermo Fisher Scientific, Waltham, MA); α‐tubulin (1:5000, 66031‐1‐Ig), α‐SMA (1:3000, 14395‐1‐Ig), GAPDH (1:10 000, 60004‐1‐Ig), histone 3 (1:1000, 17168‐1‐AP), TFAM (1:1000, 22586‐1‐AP), and ubiquitin (Ub, 1:1000, 10201‐2‐AP) from Proteintech (Wuhan, China); FN (1:2000, F3648, Sigma‐Aldrich, Darmstadt, Germany); TOM20 (1:2000, ab186735), p16^INK4A^ (1:2000, ab108349), and cullin 4B (1:5000, ab76470) from Abcam (Cambridge, England); and PGC1α (1:1000, AB3242, Millipore, Darmstadt, Germany).

### DNA Isolation and mtDNA Copies Identification

Following the manufacturer's instructions, total DNA was isolated from mTECs and renal tissues using a TIANamp Genomic DNA kit (DP304, TIGENGEN, Beijing, China). The DNA was subsequently used for qPCR. Copies of mitochondrial cytochrome c oxidase subunit 2 (*COX2*) DNA were used to represent the copies of mtDNA, and the relative abundance of mtDNA was normalized to nuclear ribosomal protein 18 (*RSP18*). The primer sequence is displayed in Table S3 (Supporting Information).

### ATP Measurement

Cellular ATP levels were measured using an ATP assay kit (S0026, Beyotime, Shanghai, China). Briefly, cells were lysed in the ATP lysis buffer and centrifuged at 12 000 rpm for 5 min at 4 °C. The supernatant was collected, and 20 µL supernatant was mixed with 100 µL ATP detection working fluid. The ATP concentration was measured by reading the luminescence on a microplate luminometer (Biotek, Milan, Italy) and converted based on the ATP standard curve. Finally, the ATP concentration was normalized to nmol mg^−1^ protein.

### SA‐β‐gal Staining

According to the manufacturer's instructions, the cultured cells and frozen renal slices (5 µm) were fixed and stained using an SA‐β‐gal staining kit (C0602, Beyotime, Shanghai, China).

### Analysis of Mitochondrial Morphology

Cells were incubated with 20 nmol L^−1^ MitoTracker Red CMXRos (M7512, Thermo Fisher Scientific, Waltham, MA) for 15 min at 37 °C. Next, the cells were washed with PBS and incubated with 1 drop mL^−1^ NucBlue Live ReadyProbes Reagent (Hoechst, R37605, Thermo Fisher Scientific, Waltham, MA). The stained cells were viewed at 600× magnification using a confocal microscope (NCF950, NOVEL, Ningbo, China).

Using a previously reported method,^[^ ^44^ ^]^ mitochondrial morphological parameters, including individual mitochondria, mitochondrial networks, and mitochondrial length, were quantified using MiNa, a semi‐automated ImageJ plugin. Each experiment was repeated three times and at least 10 cells were analyzed per replicate.

### OCR Measurement

Fresh renal tissues were shredded into small pieces, the cultured cells were suspended by trypsinization, and the samples were dissolved in 2 mL PBS with 25 mmol L^−1^ glucose, 1 mmol L^−1^ pyruvate, and 2% bovine serum albumin. The OCR was examined using a liquid‐phase oxygen measurement system (Oxygenph+, Hansatech, Shandong, China) according to the manufacturer's instructions. Oxygen consumption was recorded until 1 min using the OxyTrace system, and the data were normalized to the total protein.

### Serum Biochemical Assays

Blood samples were centrifuged to collect the serum. BUN and creatinine levels were evaluated using the urea nitrogen assay kit (C013‐2‐1) and creatinine assay kit (C011‐2‐1), respectively, from Nanjing Jiancheng Bioengineering Institute (Nanjing, China).

### Immunofluorescence Staining

Paraffin‐embedded mouse kidney tissues were subjected to immunofluorescence as the established protocols. The mouse kidney slides were incubated with anti‐AhR (1:50, MA1‐514, Thermo Fisher Scientific, Waltham, MA), anti‐Megalin (1:50, sc‐515772, Santa Cruz, Dallas, Texas), anti‐NCC (1:200, ab95302, Abcam, Cambridge, England), anti‐p21 (1:20, sc‐6246, Santa Cruz, Dallas, Texas), or p16^INK4A^ (1:100, ab108349, Abcam, Cambridge, England) antibodies. MTECs were seeded in a confocal dish (Costar) and fixed with 4% paraformaldehyde at room temperature for 20 min. Fixed cells were permeabilized with 0.5% Triton X‐100 for 10 min and blocked with 5% goat serum for 1 h. Then, the cells were incubated overnight with specific anti‐AhR (1:50, MA1‐514, Waltham, MA). Primary TECs were incubated with anti‐Megalin (1:50) or anti‐E‐cadherin (1:100, 610 181, BD Biosciences, New Jersey) antibodies. Secondary antibodies and 4′,6‐diamidino‐2‐phenylindole were used (Beyotime, Shanghai, China) to visualize primary antibodies and nuclei, respectively. Slides and cells were visualized under a confocal microscope (Leica, Wetzlar, Germany).

### Isolation of Cytoplasmic and Nuclear Protein

Cytoplasmic and nuclear proteins were isolated using an extraction kit (P0028, Beyotime, Shanghai, China) according to the manufacturer's instructions. Briefly, cells were lysed in cytoplasmic extraction buffer and centrifuged to collect the supernatant fraction as cytoplasmic protein. The sediment was resuspended in nuclear extraction buffer for 30 min at 4 °C, and then centrifugated to collect nuclear fraction in supernatant.

### Serum IS Concentration Measurement

The serum IS concentration was measured using liquid chromatography/mass spectrometry as described previously.^[^ ^45^ ^]^ In brief, 100 µL serum was mixed with 900 µL cold aqueous methanol (80% v v^−1^) and placed at 20 °C for 1 h. The solution was centrifuged at 4 °C for 15 min at a speed of 12 000 g. Following reconstitution with 80 µL water, the supernatant was evaporated using a nitrogen stream. Next, 20 µL of solution was injected and detected using an API 6500 Qtrap Mass Spectrograph (Thermo Fisher Scientific, Waltham, MA). A standard curve was built up by dissolving IS at different concentrations and detected using the same protocol.

### IP and Ubiquitination Assay

MTECs were transfected with the indicated vectors for 12 h, then treated with 1000 µmol L^−1^ IS for 36 h, followed by MG132 treatment for 4 h before being harvested. Then, the cells were lysed using Western blot and IP lysis buffer (P0013, Beyotime, Shanghai, China) containing a protease cocktail (0 469 313 2001, Roche, Basel, Switzerland) and phenylmethylsulfonyl fluoride (ST505, Beyotime, Shanghai, China). The extract was centrifuged at 12 000 rpm for 10 min at 4 °C to obtain the supernatant. 800–1000 µg total protein was hybridized with 3–5 µg primary antibodies by vertical rotation at 4 °C overnight. Then, the complex was incubated with Protein A/G PLUS‐Agarose (sc‐2003, Santa Cruz, Dallas, Texas) at 4 °C for 4 h. Finally, the precipitate was washed with PBS for six times. The protein was resuspended with 30 µL 1× SDS loading buffer followed by Western blot.

### Statistical Analysis

The normal distribution test of data was performed using the Shapiro‐Wilks normality test. For comparisons of two groups of normally distributed data, two‐tailed unpaired Student's *t* test was used for data of equal variances, or with Welch's correction if equal variances are not assumed by an *F* test. For comparisons of more than two groups, the Brown‐Forsythe test was used to assess similar variances, followed by one‐way ANOVA or two‐way ANOVA with Tukey's multiple comparisons test. The Mann‐Whitney test was used for the two groups of data that did not conform to the normal distribution. Spearman's correlation analysis was used to assess the correlation between AhR mRNA level and eGFR. Statistical analysis of the data was carried out using GraphPad Prism software 8 (La Jolla, CA) and data were shown as mean ± SD. Sample size (*n*) for each statistical analysis is indicated in each figure legend. *P* < 0.05 was considered to be statistically significant.

### Ethics Approval

All animal experimental protocols and procedures complied with the Guidance for Care and Use of Laboratory Animals of Fudan University and were approved by the Ethics Committee for Experimental Research of Shanghai Medical College, Fudan University, Shanghai, China (20200306‐097).

## Conflict of Interest

The authors declare no conflict of interest

## Author Contributions

H.X. and N.Y. contributed equally to this work. H.X. performed conceptualization, data curation, and wrote and reviewed the draft. N.Y. and X.S. performed data curation. L.L. performed data curation and methodology. J.L., X.W., and H.G. performed formal analysis. L.Z., J.L., and H.W. performed methodology. C.Y. and W.Z. performed supervision. L.L. performed supervision and wrote and reviewed the draft.

## Supporting information

Supporting Information

## Data Availability

The data that support the findings of this study are available from the corresponding author upon reasonable request.

## References

[1] A. J. Collins , R. N. Foley , B. Chavers , D. Gilbertson , C. Herzog , K. Johansen , B. Kasiske , N. Kutner , J. Liu , W. St Peter , H. Guo , S. Gustafson , B. Heubner , K. Lamb , S. Li , S. Li , Y. Peng , Y. Qiu , T. Roberts , M. Skeans , J. Snyder , C. Solid , B. Thompson , C. Wang , E. Weinhandl , D. Zaun , C. Arko , S. C. Chen , F. Daniels , J. Ebben , et al., Am. J. Kidney Dis. 2012, 59, 1.22177443

[2] M. H. Docherty , E. D. O'Sullivan , J. V. Bonventre , D. A. Ferenbach , J. Am. Soc. Nephrol. 2019, 30, 726. 31000567 10.1681/ASN.2018121251 PMC6493983

[3] I. Sturmlechner , M. Durik , C. J. Sieben , D. J. Baker , J. M. van Deursen , Nat. Rev. Nephrol. 2017, 13, 77. 28029153 10.1038/nrneph.2016.183

[4] C. Luo , S. Zhou , Z. Zhou , Y. Liu , L. Yang , J. Liu , Y. Zhang , H. Li , Y. Liu , F. F. Hou , L. Zhou , J. Am. Soc. Nephrol. 2018, 29, 1238. 29440280 10.1681/ASN.2017050574 PMC5875944

[5] C. Jia , C. Ke‐Hong , X. Fei , D. Huan‐Zi , Y. Jie , W. Li‐Ming , W. Xiao‐Yue , Z. Jian‐Guo , H. Ya‐Ni , Kidney Int. 2020, 98, 645. 32739204 10.1016/j.kint.2020.03.026

[6] K. Wang , B. Kestenbaum , Clin. J. Am. Soc. Nephrol. 2018, 13, 1291. 29490976 10.2215/CJN.12001017 PMC6086711

[7] P. Bhargava , R. G. Schnellmann , Nat. Rev. Nephrol. 2017, 13, 629. 28804120 10.1038/nrneph.2017.107 PMC5965678

[8] J. Miao , J. Liu , J. Niu , Y. Zhang , W. Shen , C. Luo , Y. Liu , C. Li , H. Li , P. Yang , Y. Liu , F. F. Hou , L. Zhou , Aging Cell 2019, 18, 13004. 10.1111/acel.13004 PMC6718575 31318148

[9] X. Yu , M. Xu , X. Meng , S. Li , Q. Liu , M. Bai , R. You , S. Huang , L. Yang , Y. Zhang , Z. Jia , A. Zhang , Sci Transl Med. 2020, 12, eaay7591. 32404507 10.1126/scitranslmed.aay7591

[10] A. M. Hall , C. D. Schuh , Curr. Opin. Nephrol. Hypertens. 2016, 25, 355. 27166518 10.1097/MNH.0000000000000228

[11] K. W. Chung , P. Dhillon , S. Huang , X. Sheng , R. Shrestha , C. Qiu , B. A. Kaufman , J. Park , L. Pei , J. Baur , M. Palmer , K. Susztak , Cell Metab. 2019, 30, 784. 31474566 10.1016/j.cmet.2019.08.003 PMC7054893

[12] J. D. Ravid , M. H. Kamel , V. C. Chitalia , Nat Rev Nephrol 2021, 17, 402. 33758363 10.1038/s41581-021-00408-4

[13] C. Y. Sun , S. C. Chang , M. S. Wu , Kidney Int. 2012, 81, 640. 22237753 10.1038/ki.2011.445 PMC3306006

[14] J. Lv , J. Chen , M. Wang , F. Yan , Aging (Albany NY) 2020, 12, 9139. 32464602 10.18632/aging.103183 PMC7288965

[15] E. C. Henry , S. L. Welle , T. A. Gasiewicz , Toxicol. Sci. 2010, 114, 90. 19933214 10.1093/toxsci/kfp285 PMC2819971

[16] Y. Shi , Z. Zeng , J. Yu , B. Tang , R. Tang , R. Xiao , Pharmacol. Res. 2020, 160, 105180. 32877693 10.1016/j.phrs.2020.105180

[17] J. C. Schroeder , B. C. Dinatale , I. A. Murray , C. A. Flaveny , Q. Liu , E. M. Laurenzana , J. M. Lin , S. C. Strom , C. J. Omiecinski , S. Amin , G. H. Perdew , Biochemistry 2010, 49, 393. 20000589 10.1021/bi901786x PMC2805781

[18] S. Heath‐Pagliuso , W. J. Rogers , K. Tullis , S. D. Seidel , P. H. Cenijn , A. Brouwer , M. S. Denison , Biochemistry 1998, 37, 11508. 9708986 10.1021/bi980087p

[19] O. Hankinson , Annu. Rev. Pharmacol. Toxicol. 1995, 35, 307. 7598497 10.1146/annurev.pa.35.040195.001515

[20] J. S. Brito , N. A. Borges , M. Esgalhado , D. C. Magliano , C. O. Soulage , D. Mafra , Nephron 2017, 137, 1. 10.1159/000476074 28490014

[21] F. Ohtake , A. Baba , I. Takada , M. Okada , K. Iwasaki , H. Miki , S. Takahashi , A. Kouzmenko , K. Nohara , T. Chiba , Y. Fujii‐Kuriyama , S. Kato , Nature 2007, 446, 562. 17392787 10.1038/nature05683

[22] J. A. Walker , S. Richards , M. E. Belghasem , N. Arinze , S. B. Yoo , J. Y. Tashjian , S. A. Whelan , N. Lee , V. B. Kolachalama , J. Francis , K. Ravid , D. Sherr , V. C. Chitalia , Kidney Int. 2020, 97, 538. 31932072 10.1016/j.kint.2019.09.029 PMC9721455

[23] H. Y. Xie , N. H. Yang , C. Yu , L. M. Lu , Cell. Mol. Biol. Lett. 2024, 29, 38. 38491448 10.1186/s11658-024-00550-4 PMC10943832

[24] S. H. Kim , E. C. Henry , D. K. Kim , Y. H. Kim , K. J. Shin , M. S. Han , T. G. Lee , J. K. Kang , T. A. Gasiewicz , S. H. Ryu , P. G. Suh , Mol. Pharmacol. 2006, 69, 1871. 16540597 10.1124/mol.105.021832

[25] B. Zhao , D. E. Degroot , A. Hayashi , G. He , M. S. Denison , Toxicol. Sci. 2010, 117, 393. 20634293 10.1093/toxsci/kfq217 PMC2940411

[26] Q. Zheng , H. Liu , H. Zhang , Y. Han , J. Yuan , T. Wang , Y. Gao , Z. Li , Adv. Sci. (Weinh) 2023, 10, 2300758. 37202595 10.1002/advs.202300758 PMC10401119

[27] S. H. Seok , W. Lee , L. Jiang , K. Molugu , A. Zheng , Y. Li , S. Park , C. A. Bradfield , Y. Xing , Proc. Natl. Acad. Sci. USA 2017, 114, 5431. 28396409 10.1073/pnas.1617035114 PMC5448172

[28] S. Shivanna , K. Kolandaivelu , M. Shashar , M. Belghasim , L. Al‐Rabadi , M. Balcells , A. Zhang , J. Weinberg , J. Francis , M. P. Pollastri , E. R. Edelman , D. H. Sherr , V. C. Chitalia , J. Am. Soc. Nephrol. 2016, 27, 189. 26019318 10.1681/ASN.2014121241 PMC4696580

[29] K. Bruck , V. S. Stel , G. Gambaro , S. Hallan , H. Volzke , J. Arnlov , M. Kastarinen , I. Guessous , J. Vinhas , B. Stengel , H. Brenner , J. Chudek , S. Romundstad , C. Tomson , A. O. Gonzalez , A. K. Bello , J. Ferrieres , L. Palmieri , G. Browne , V. Capuano , W. Van Biesen , C. Zoccali , R. Gansevoort , G. Navis , D. Rothenbacher , P. M. Ferraro , D. Nitsch , C. Wanner , K. J. Jager , C. K. D. B. C. European , J. Am. Soc. Nephrol. 2016, 27, 2135. 26701975 10.1681/ASN.2015050542 PMC4926978

[30] R. Vanholder , R. De Smet , G. Glorieux , A. Argiles , U. Baurmeister , P. Brunet , W. Clark , G. Cohen , P. P. De Deyn , R. Deppisch , B. Descamps‐Latscha , T. Henle , A. Jorres , H. D. Lemke , Z. A. Massy , J. Passlick‐Deetjen , M. Rodriguez , B. Stegmayr , P. Stenvinkel , C. Tetta , C. Wanner , W. Zidek , G. European Uremic Toxin Work , Kidney Int. 2003, 63, 1934. 12675874 10.1046/j.1523-1755.2003.00924.x

[31] F. Duranton , G. Cohen , R. De Smet , M. Rodriguez , J. Jankowski , R. Vanholder , A. Argiles , G. European Uremic Toxin Work , J. Am. Soc. Nephrol. 2012, 23, 1258. 22626821 10.1681/ASN.2011121175 PMC3380651

[32] A. Adijiang , H. Shimizu , Y. Higuchi , F. Nishijima , T. Niwa , J Ren Nutr 2011, 21, 105. 21195930 10.1053/j.jrn.2010.10.020

[33] H. Shimizu , D. Bolati , A. Adijiang , G. Muteliefu , A. Enomoto , F. Nishijima , M. Dateki , T. Niwa , Am. J. Physiol. Cell Physiol. 2011, 301, C1201. 21832251 10.1152/ajpcell.00471.2010

[34] G. Muteliefu , H. Shimizu , A. Enomoto , F. Nishijima , M. Takahashi , T. Niwa , Am. J. Physiol. Cell Physiol. 2012, 303, C126. 22555846 10.1152/ajpcell.00329.2011

[35] V. Brinkmann , N. Ale‐Agha , J. Haendeler , N. Ventura , Front. Physiol. 2019, 10, 1561. 32009975 10.3389/fphys.2019.01561 PMC6971224

[36] R. C. Scarpulla , Physiol. Rev. 2008, 88, 611. 18391175 10.1152/physrev.00025.2007

[37] M. Fontecha‐Barriuso , D. Martin‐Sanchez , J. M. Martinez‐Moreno , M. Monsalve , A. M. Ramos , M. D. Sanchez‐Nino , M. Ruiz‐Ortega , A. Ortiz , A. B. Sanz , Biomolecules 2020, 10, 347. 32102312 10.3390/biom10020347 PMC7072614

[38] W. J. Lee , S. H. Liu , C. K. Chiang , S. Y. Lin , K. W. Liang , C. H. Chen , H. R. Tien , P. H. Chen , J. P. Wu , Y. C. Tsai , D. W. Lai , Y. C. Chang , W. H. Sheu , M. L. Sheu , Antioxid. Redox Signal 2016, 24, 217. 26415004 10.1089/ars.2015.6310

[39] K. Kawajiri , Y. Kobayashi , F. Ohtake , T. Ikuta , Y. Matsushima , J. Mimura , S. Pettersson , R. S. Pollenz , T. Sakaki , T. Hirokawa , T. Akiyama , M. Kurosumi , L. Poellinger , S. Kato , Y. Fujii‐Kuriyama , Proc. Natl. Acad. Sci. USA 2009, 106, 13481. 19651607 10.1073/pnas.0902132106 PMC2726415

[40] W. C. Liu , J. F. Shyu , P. S. Lim , T. C. Fang , C. L. Lu , C. M. Zheng , Y. C. Hou , C. C. Wu , Y. F. Lin , K. C. Lu , Int J Mol Sci. 2020, 21, 3486. 32429048 10.3390/ijms21103486 PMC7278944

[41] Z. Ma , Z. Li , Y. Mao , J. Ye , Z. Liu , Y. Wang , C. Wei , J. Cui , Z. Liu , X. Liang , Nat. Commun. 2023, 14, 5415. 37670034 10.1038/s41467-023-41218-5 PMC10480448

[42] L. Ouyang , C. Yu , Z. Xie , X. Su , Z. Xu , P. Song , J. Li , H. Huang , Y. Ding , M. H. Zou , Circulation 2022, 145, 1784. 35582948 10.1161/CIRCULATIONAHA.121.057868 PMC9197997

[43] L. L. Lv , Y. Feng , M. Wu , B. Wang , Z. L. Li , X. Zhong , W. J. Wu , J. Chen , H. F. Ni , T. T. Tang , R. N. Tang , H. Y. Lan , B. C. Liu , Cell Death Differ. 2020, 27, 210. 31097789 10.1038/s41418-019-0349-y PMC7206053

[44] A. J. Valente , L. A. Maddalena , E. L. Robb , F. Moradi , J. A. Stuart , Acta Histochem. 2017, 119, 315. 28314612 10.1016/j.acthis.2017.03.001

[45] N. Fabresse , I. Uteem , E. Lamy , Z. Massy , I. A. Larabi , J. C. Alvarez , Clin. Chim. Acta 2020, 507, 228. 32371217 10.1016/j.cca.2020.04.032

