# Supplementary material for: Uremic Toxin Receptor AhR Facilitates Renal Senescence and Fibrosis via Suppressing Mitochondrial Biogenesis
Source: Adv Sci (Weinh). 2024 Jun 28;11(33):2402066. doi: 10.1002/advs.202402066 (PMC11434102; doi:10.1002/advs.202402066)
Supplement: Supplementary file 1 — Supporting Information [file ADVS-11-2402066-s001.docx]

Supporting Information

Uremic Toxin Receptor AhR Facilitates Renal Senescence and Fibrosis via Suppressing Mitochondrial Biogenesis

*Hongyan Xie, Ninghao Yang, Li Lu, Xi’ang Sun, Jingyao Li, Xin Wang, Hengjiang Guo, Li Zhou, Jun Liu, Huijuan Wu, Chen Yu*^*^*, Wei Zhang*^*^*, and Limin Lu*^*^*.*


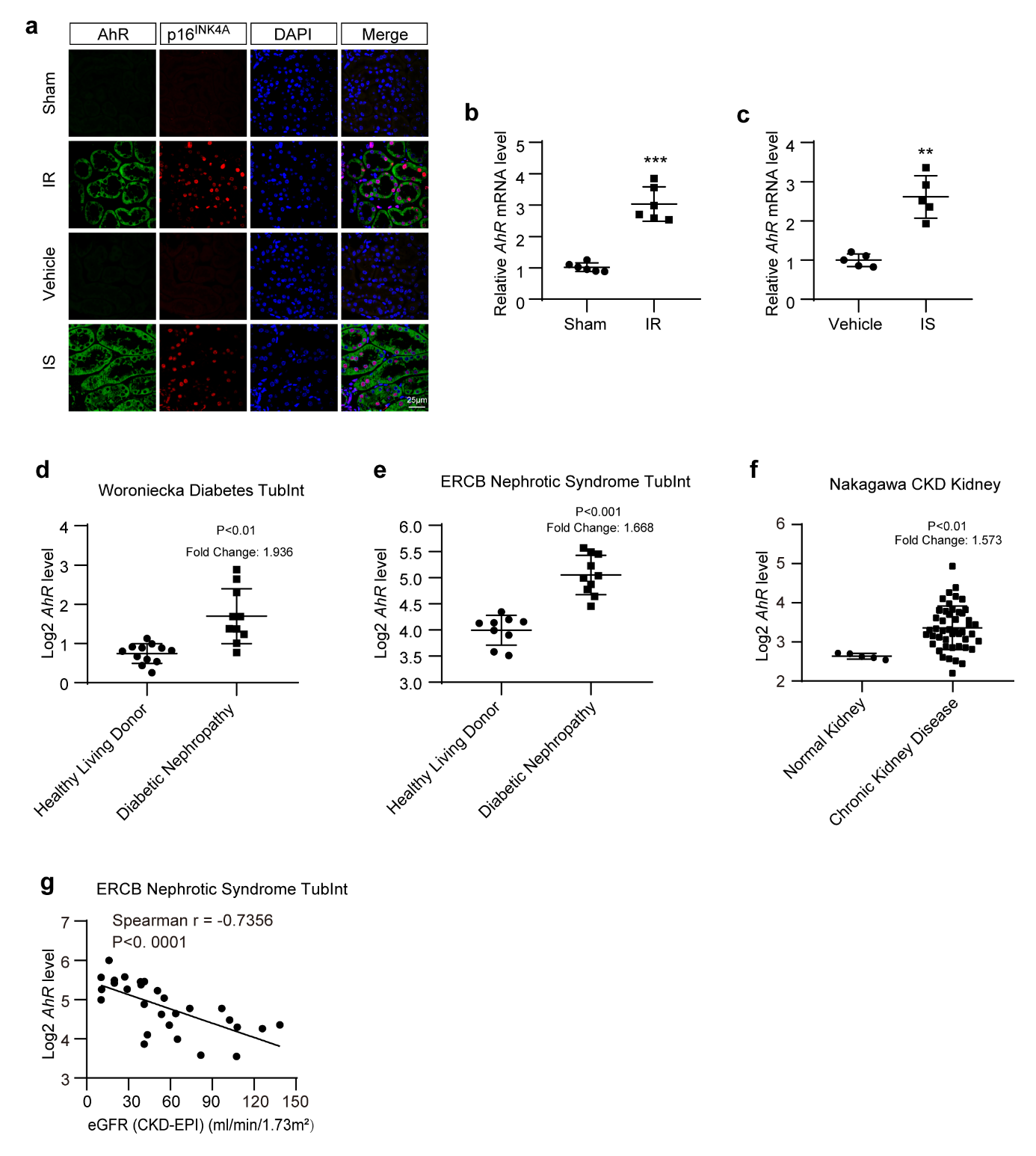


**Figure S1.** *AhR* mRNA level was upregulated in the kidneys of CKD mice and patients. (a) Co-immunofluorescence of AhR and p16^INK4A^ in mouse kidneys after IR or IS treatment. Scale bar, 25 μm. (b and c) The mRNA level of *AhR* in the kidneys of IR mice (b) and IS mice (c). ^**^*P* < 0.01 versus vehicle group (*n*=5), ^***^*P* < 0.001 versus sham group (*n*= 6). (d-f) The mRNA level of *AhR* in the kidneys of healthy controls (*n*=12) or patients with diabetic nephropathy (*n*=10) from Woroniecka Diabetes Tublnt data set (d), healthy controls (*n*=9) or patients with diabetic nephropathy (*n*=10) from ERCB Nephrotic Syndrome Tublnt data set (e), and healthy controls (*n*=5) or patients with chronic kidney disease (*n*=48) from Nakagawa CKD Kidney data set (f). (g) Linear regression analysis of the correlation between *AhR* mRNA level and estimated glomerular filtration rate (eGFR, *n*=28). Data were shown as mean ± SD. Statistical analysis was performed by two-tailed unpaired Student’s *t*-test (e), two-tailed unpaired Welch’ *t*-test (b, c, d), Mann-Whitney test (f), and Spearman correlation test (g).


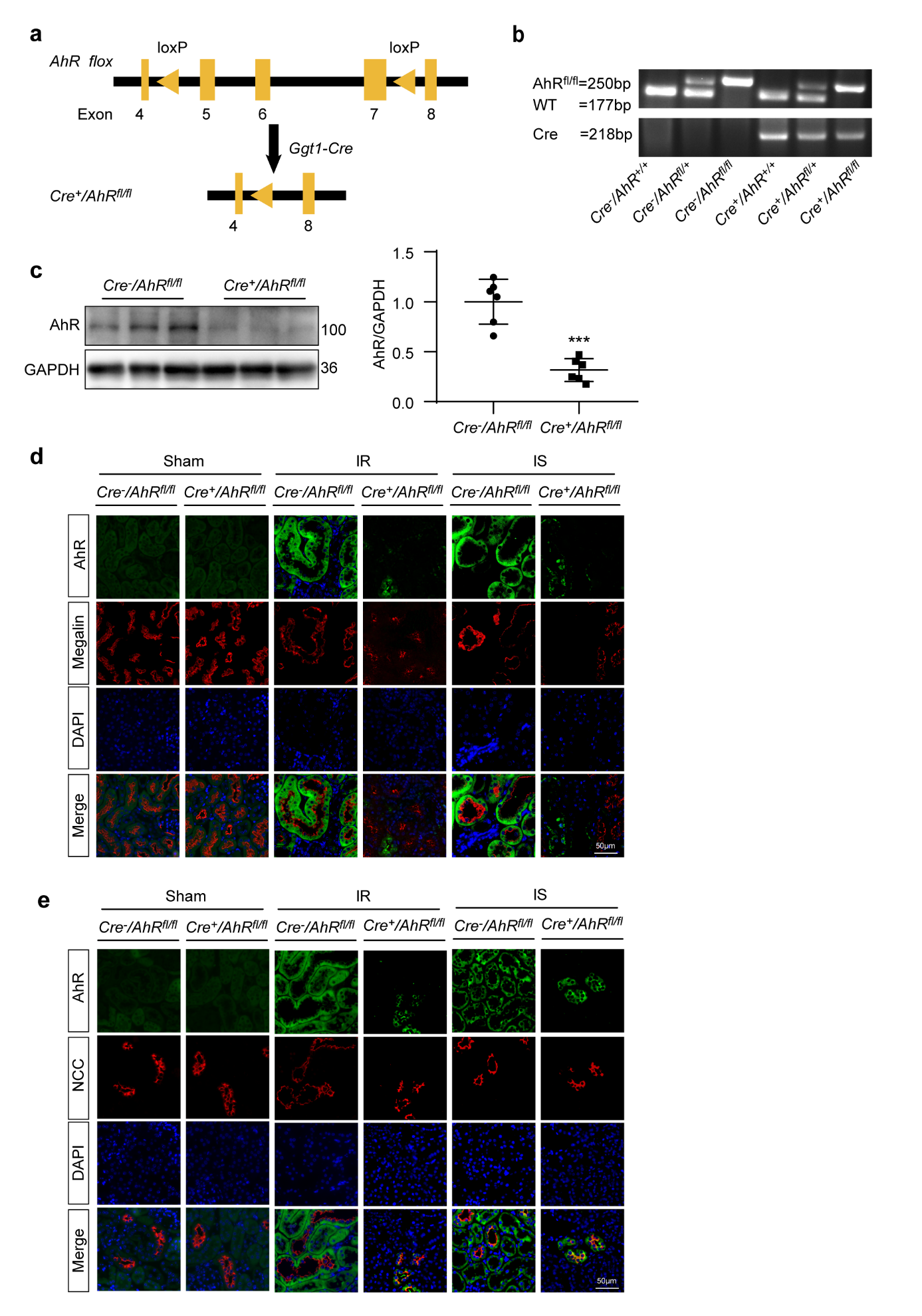


**Figure S2.** Generation and identification of tubular epithelial cell (TEC)-specific *AhR* knockout (*Cre^+^AhR^fl/fl^*) mice. (a) *Cre^+^AhR^fl/fl^* mice were generated by crossbreeding transgenic *AhR^fl/fl^* mice with *Ggt1-Cre* mice driven by proximal tubular epithelial cellular *Ggt1* promoter. (b) Genotype identification by PCR amplification of DNA isolated from 2-week-old mice tail tissue. (c) Western blot images and quantitative analysis of AhR level in the kidneys from different groups of mice. ^***^*P* < 0.001 versus *Cre^-^AhR^fl/fl^* group (*n*=6). (d and e) Co-immunofluorescence micrographs of AhR and Megalin (a marker of proximal tubules; d) or NCC (a marker of distal tubules; e) in mouse kidneys after IR or IS treatment. Scale bar, 50 μm. Data were shown as mean ± SD. Statistical analysis was performed by two-tailed unpaired Student’s *t*-test (c).


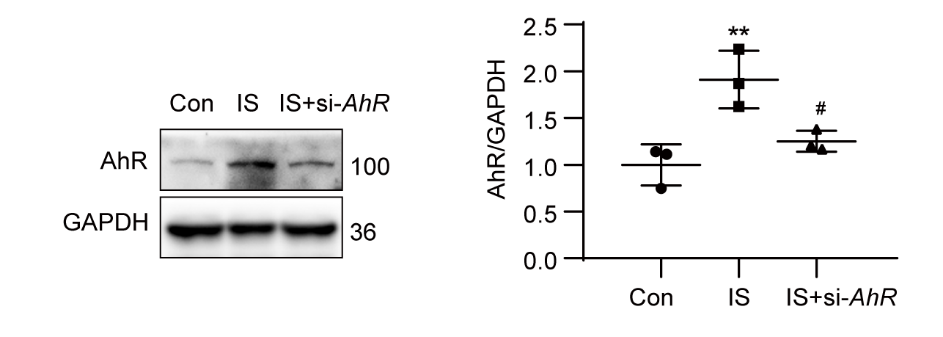


**Figure S3.** Validation of AhR knockdown efficiency in mouse tubular epithelial cells (mTECs). The mTECs were transfected with *AhR* siRNA (si-*AhR*) for 12 h and then treated with IS (1000 μmol L^-1^) for an additional 36 h. Western blot images and quantitative data of AhR in mTECs. ^**^*P* < 0.01 versus Con group, ^#^*P* < 0.05 versus IS group (*n*=3). Data were shown as mean ± SD. Statistical analysis was performed by one-way ANOVA with Tukey’s multiple comparisons test.

*
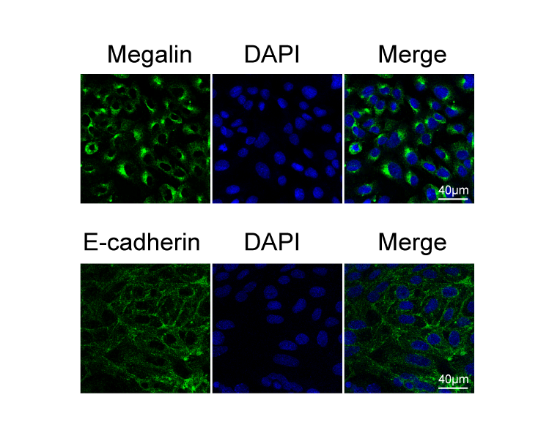
*

**Figure S4.** Validation of primary proximal TECs. Immunofluorescence of Megalin and E-cadherin in primary proximal TECs. Scale bar, 40 μm.


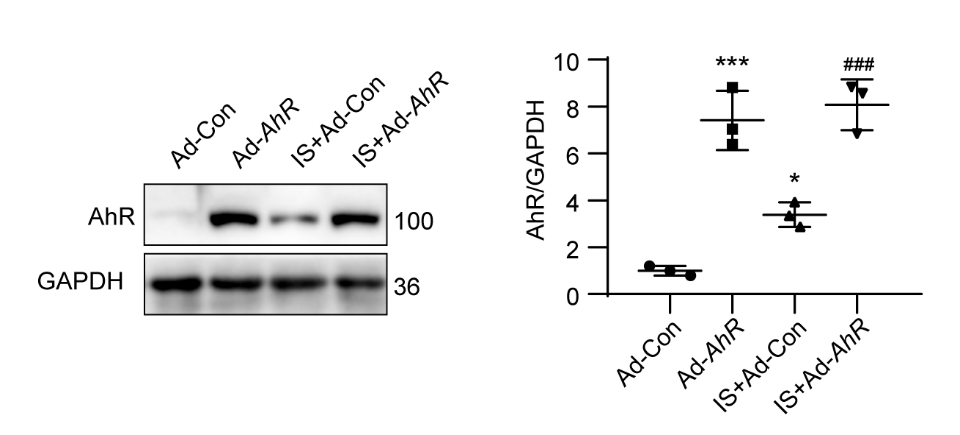


**Figure S5.** Validation of AhR overexpression efficiency in mTECs. Western blot images and quantitative analysis of AhR in mTECs. ^*^*P* < 0.05, ^***^*P* < 0.001 versus empty adenovirus vector (Ad-Con) group, ^###^*P* < 0.001 versus IS+Ad-Con group (*n*=3). Data were shown as mean ± SD. Statistical analysis was performed by two-way ANOVA with Tukey’s multiple comparisons test.


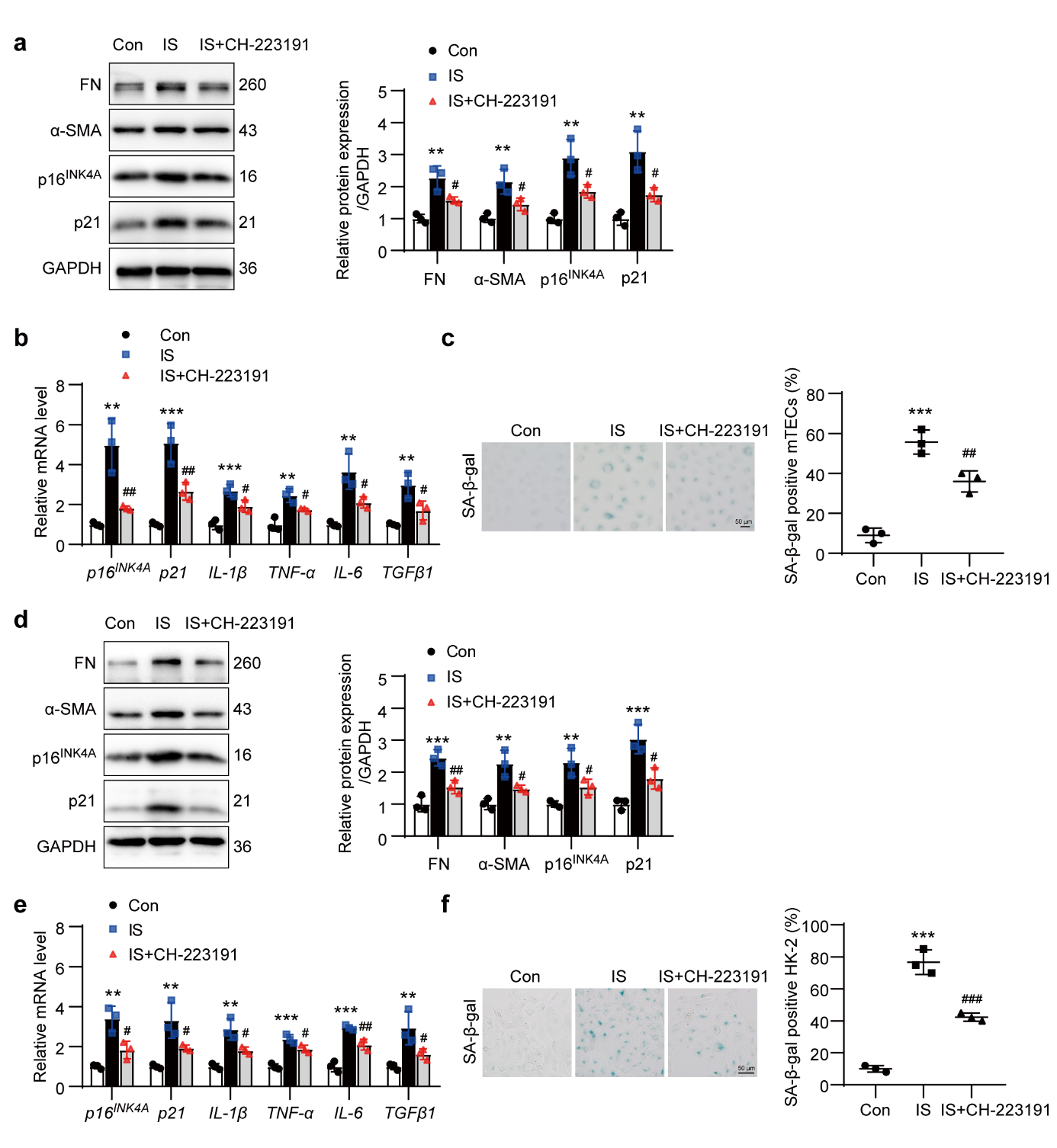


**Figure S6.** Inhibition of AhR activation significantly alleviated IS-induced cell senescence and extracellular matrix (ECM) production in mTECs and human proximal tubular epithelial cells (HK-2). The mTECs were pretreated with CH-223191 (10 μmol L^-1^) for 0.5 h and then treated with IS (1000 μmol L^-1^) for 36 h. (a) Western blot images and quantitative data of FN, α-SMA, p16^INK4A^ and p21 in mTECs. ^**^*P* < 0.01 versus Con group, ^#^*P* < 0.05 versus IS group (*n*=3). (b) The relative mRNA abundance of *p16^INK4A^*, *p21*, *IL-1β*, *TNF-α*, *IL-6* and *TGFβ1* in mTECs. ^**^*P* < 0.01, ^***^*P* < 0.001 versus Con group, ^#^*P* < 0.05, ^##^*P* < 0.01 versus IS group (*n*=3). (c) The SA-β-gal staining micrographs and quantitative data showing mTEC senescence. Scale bar, 50 μm. ^***^*P* < 0.001 versus Con group, ^##^*P* < 0.01 versus IS group (*n*=3). HK-2 cells were pretreated with CH-223191 (10 μmol L^-1^) for 0.5 h and then treated with IS (1000 μmol L^-1^) for 36 h. (d) Western blot images and quantitative data of FN, α-SMA, p16^INK4A^ and p21 in HK-2 cells. ^**^*P* < 0.01, ^***^*P* < 0.001 versus Con group, ^#^*P* < 0.05, ^##^*P* < 0.01 versus IS group (*n*=3). (e) The relative mRNA abundance of *p16^INK4A^*, *p21*, *IL-1β*, *TNF-α*, *IL-6* and *TGFβ1* in HK-2 cells. ^**^*P* < 0.01, ^***^*P* < 0.001 versus Con group, ^#^*P* < 0.05, ^##^*P* < 0.01 versus IS group (*n*=3). (f) The SA-β-gal staining micrographs and quantitative data showing HK-2 senescence. Scale bar, 50 μm. ^***^*P* < 0.001 versus Con group, ^###^*P* < 0.001 versus IS group (*n*=3). Data were shown as mean ± SD. Statistical analysis was performed by one-way ANOVA with Tukey’s multiple comparisons test.


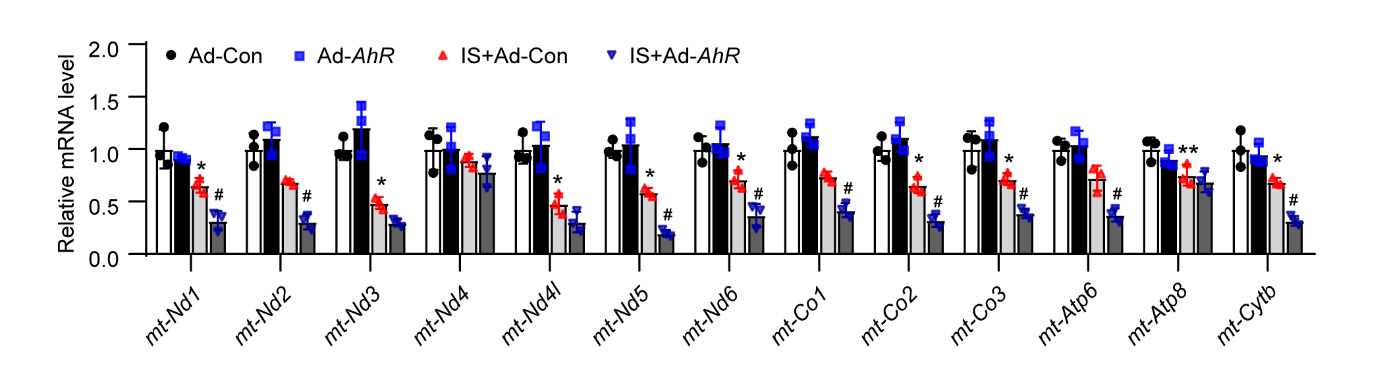


**Figure S7.** AhR promoted the suppression of mitochondrial biogenesis in mTECs. qPCR assessing the mRNA levels of 13 mtDNA in mTECs. ^*^*P* < 0.05, ^**^*P* < 0.01 versus Ad-Con group, ^#^*P* < 0.05 versus IS+Ad-Con group (*n*=3). Data were shown as mean ± SD. Statistical analysis was performed by two-way ANOVA with Tukey’s multiple comparisons test.


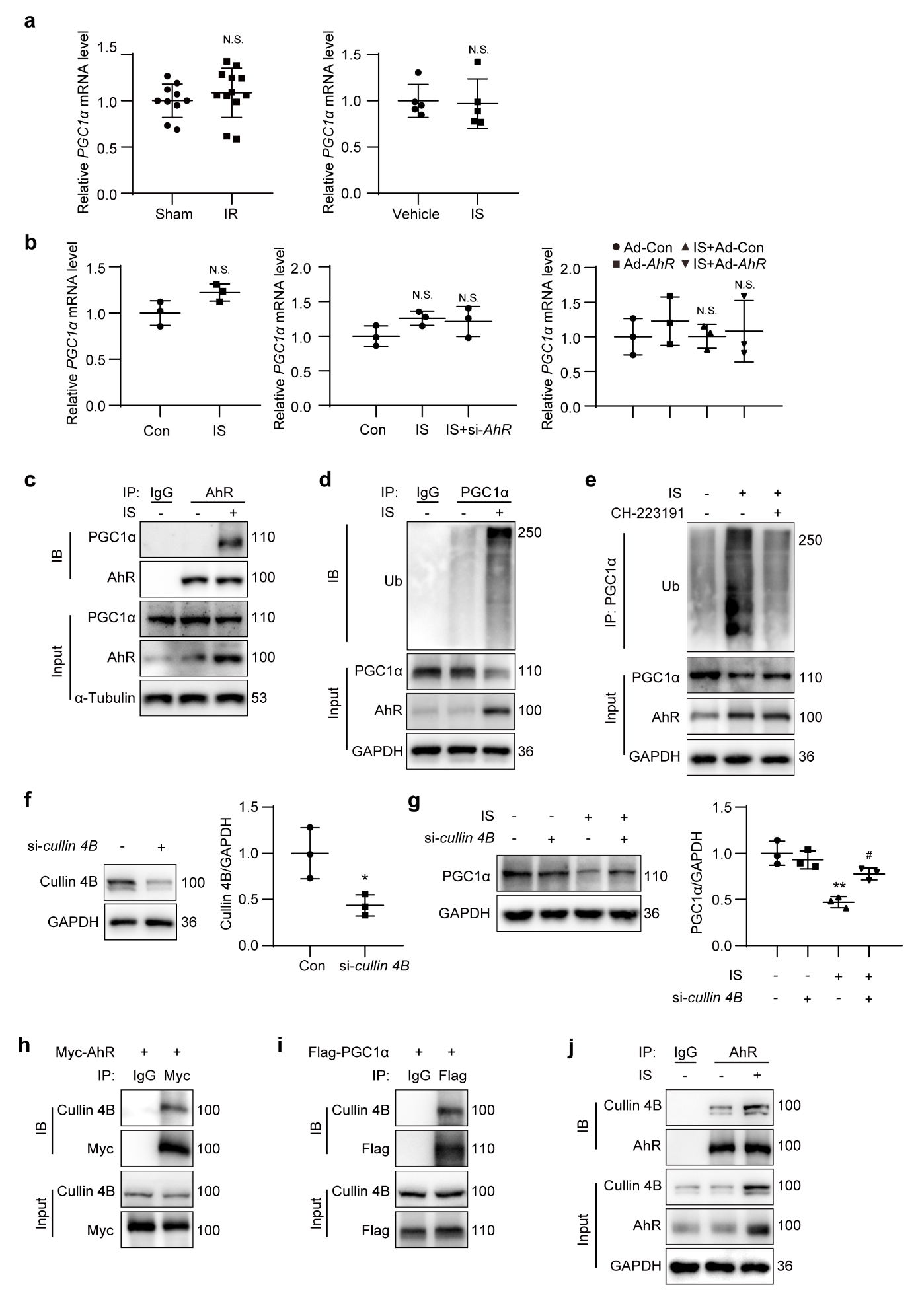


**Figure S8.** AhR promoted PGC1α ubiquitination. (a) qPCR detecting the mRNA level of *PGC1α* in the kidneys of IR- or IS-treated mice. N.S., no significant difference versus sham or vehicle mice (*n*=5-12). (b) qPCR detecting the mRNA level of *PGC1α* in mTECs transfected with *AhR* siRNA (si-*AhR*) or adenovirus carrying AhR gene (Ad-*AhR*) with IS stimulation (1000 μmol L^-1^) for 48 h. N.S., no significant difference versus Con, IS, Ad-Con group or IS+Ad-Con group (*n*=3). (c) Co-IP of AhR and PGC1α in mTECs subjected to 36-h IS treatment (1000 μmol L^-1^) in the presence of MG132 (20 μmol L^-1^) for 4 h. The lysates were immunoprecipitated with control IgG or anti-AhR antibodies, followed by Western blot with anti-PGC1α antibody (*n*=3). (d) IP analysis of PGC1α ubiquitination in mTECs in the presence of IS (1000 μmol L^-1^) for 36 h. MG132 (20 μmol L^-1^) was added to the medium 4 h before cell harvest. The lysates were immunoprecipitated with control IgG or anti-PGC1α antibodies, followed by Western blot with anti-ubiquitin (Ub) antibody (*n*=3). (e) IP analysis of PGC1α ubiquitination in HK-2 treated with AhR inhibitor CH-223191 (10 μmol L^-1^) in the presence of IS (1000 μmol L^-1^) for 48 h. MG132 (20 μmol L^-1^) was added to the medium 4 h before cell harvest. The lysates were immunoprecipitated with anti-PGC1α antibody, followed by Western blot with anti-ubiquitin (Ub) antibody (*n*=3). (f) Western blot images and quantitative data of cullin 4B in mTECs transfected with *cullin 4B* siRNA (si-*cullin 4B*) for 48 h. ^*^*P* < 0.05 versus Con group (*n*=3). (g) Western blot images and quantitative data detecting the protein level of PGC1α in mTECs transfected with si-*cullin 4B* for 12 h and then stimulated with IS (1000 μmol L^-1^) for an additional 36 h. ^**^*P* < 0.01 versus Con group, ^#^*P* < 0.05 versus IS group (*n*=3). (h and i) Co-IP of Myc or Flag with cullin 4B in HEK293T cells transfected with plasmids encoding Myc-tagged AhR WT (h) or Flag-tagged PGC1α (i) in the presence of IS (1000 μmol L^-1^) for 48 h. The lysates were immunoprecipitated with control IgG, anti-Myc, or anti-Flag antibodies, followed by Western blot with anti-cullin 4B antibody (*n*=3). (j) Co-IP of AhR and cullin 4B in mTECs in the presence of IS (1000 μmol L^-1^) for 36 h. The lysates were immunoprecipitated with control IgG or anti-AhR antibodies, followed by Western blot with anti-cullin 4B antibody (*n*=3). Data were shown as mean ± SD. Statistical analysis was performed by two-tailed unpaired Student’s *t*-test (a, the left panel of b, f), one-way ANOVA with Tukey’s multiple comparisons test (the middle panel of b), and two-way ANOVA with Tukey’s multiple comparisons test (the right panel of b, g).

**Table S1.** The primer sequence used for genotyping.

| Gene | Primer Sequence 5’ to 3’  Forward Reverse | |
| --- | --- | --- |
| *AhR flox* | AACTATGTAACTGATGCACCAAGG | ACCCTATCTCATCCATGAATCCC |
| *Ggt1-Cre* | CAGCCTGCTCTAACGGTTTC | CAGGTTCTTGCGAACCTCAT |

**Table S2.** The primer sequence used for qPCR.

| Mouse  gene | Primer Sequence 5’ to 3’  Forward Reverse | |
| --- | --- | --- |
| *AhR* | GACCACTGACGGATGAAGAAGG | CAGGGCTTGAAGGAGGACAC |
| *p16^INK4A^* | CGCAGGTTCTTGGTCACTGT | TGTTCACGAAAGCCAGAGCG |
| *FN* | TGGGAGCATTGTTGTGTCT | AGCGGTGTCACTACTCTGT |
| *COL Ⅰ* | TGACTGGAAGAGCGGAGAG | GACGGCTGAGTAGGGAACA |
| *α-SMA* | GGAGAAAATGACCCAGATT | GAGTCCAGCACAATACCAG |
| *p21* | GGCCTTGTCGCTGTCTTGCACT | GAGAGGGCAGGCAGCGTATATA |
| *IL-1β* | TGGACCTTCCAGGATGAGGACA | GTTCATCTCGGAGCCTGTAGTG |
| *TNF-α* | GATCGGTCCCCAAAGGGATG | GTGGTTTGTGAGTGTGAGGGT |
| *IL-6* | GTCCTTCCTACCCCAATTTCCA | CGCACTAGGTTTGCCGAGTA |
| *TGFβ1* | AGGGCTACCATGCCAACTTC | CCACGTAGTAGACGATGGGC |
| *PGC1α* | TCGGGAGCTGGATGGCTTGGGA | ACCAACCAGAGCAGCACACTCTA |
| *mt-Nd1* | ACACTTATTACAACCCAAGAACACAT | TCATATTATGGCTATGGGTCAGG |
| *mt-Nd2* | CCATCAACTCAATCTCACTTCTATG | GAATCCTGTTAGTGGTGGAAGG |
| *mt-Nd3* | CCATATGAATGTGGCTTCGACC | TGGTTGTTTGAATCGCTCATGG |
| *mt-Nd4* | GCTTACGCCAAACAGAT | TAGGCAGAATAGGAGTGAT |
| *mt-Nd4l* | GCCATCTACCTTCTTCA | TAGGGCTAGTCCTACAGC |
| *mt-Nd5* | GCCAACAACATATTTCAACTTTTC | ACCATCATCCAATTAGTAGAAAGGA |
| *mt-Nd6* | GGGAGATTGGTTGATGTA | ATACCCGCAAACAAAGAT |
| *mt-Co1* | CAGACCGCAACCTAAACACA | TTCTGGGTGCCCAAAGAAT |
| *mt-Co2* | GCCGACTAAATCAAGCAACA | CAATGGGCATAAAGCTATGG |
| *mt-Co3* | CGTGAAGGAACCTACCAAGG | ATTCCTGTTGGAGGTCAGCA |
| *mt-Atp6* | CCATAAATCTAAGTATAGCCATTCCAC | AGCTTTTTAGTTTGTGTCGGAAG |
| *mt-Atp8* | ACATTCCCACTGGCACC | GGGGTAATGAATGAGGC |
| *mt-Cytb* | CCCACCCCATATTAAACCCG | GAGGTATGAAGGAAAGGTATTAGGG |
| *GAPDH* | AACTTTGGCATTGTGGAAGG | ACACATTGGGGGTAGGAACA |

**Table S3.** The primer sequence used for mtDNA copies assay.

| Mouse  gene | Primer Sequence 5’ to 3’    Forward Reverse | |
| --- | --- | --- |
| *COX2* | ATAACCGAGTCGTTCTGCCAAT | TTTCAGAGCATTGGCCATAGAA |
| *RSP18* | TTCGGAACTGAGGCCATGATT | TTTCGCTCTGGTCCGTCTTG |
